# Elesesterpenes A–K: Lupane-type Triterpenoids From the Leaves of *Eleutherococcus sessiliflorus*

**DOI:** 10.3389/fchem.2021.813764

**Published:** 2022-01-24

**Authors:** Dong Han, Yan Liu, Xiao-Mao Li, Si-Yi Wang, Yan Sun, Adnan Mohammed Algradi, Hai-Dan Zou, Juan Pan, Wei Guan, Hai-Xue Kuang, Bing-You Yang

**Affiliations:** Key Laboratory of Basic and Application Research of Beiyao, Ministry of Education, Heilongjiang University of Chinese Medicine, Harbin, China

**Keywords:** *Eleutherococcus sessiliflorus*, triterpenoids, nortriterpenoid, triterpenoid glycosides, antiproliferative, anti-inflammatory

## Abstract

Elesesterpenes A–K (**1**–**11**), eleven new lupane-type triterpenoids, triterpenoid glycosides, and nortriterpenoid were isolated from the leaves of *Eleutherococcus sessiliflorus*. Their structures and relative configurations were completely elucidated by a combination of diverse methods including physical, spectroscopic data. The absolute configuration of elesesterpenes A–B (**1**–**2**) was defined by single-crystal X-ray diffraction. Meanwhile, all the isolates were evaluated for anti-inflammatory activities on lipopolysaccharide-induced nitric oxide production in BV2 microglial cells, and antiproliferative activities against human hepatoma (HepG2), human lung adenocarcinoma (A549), and human glioma cells (LN229) *in vitro*. It was found that some of them exhibited obvious anti-inflammatory activities and potent antiproliferative activities.

**GRAPHICAL ABSTRACT:**
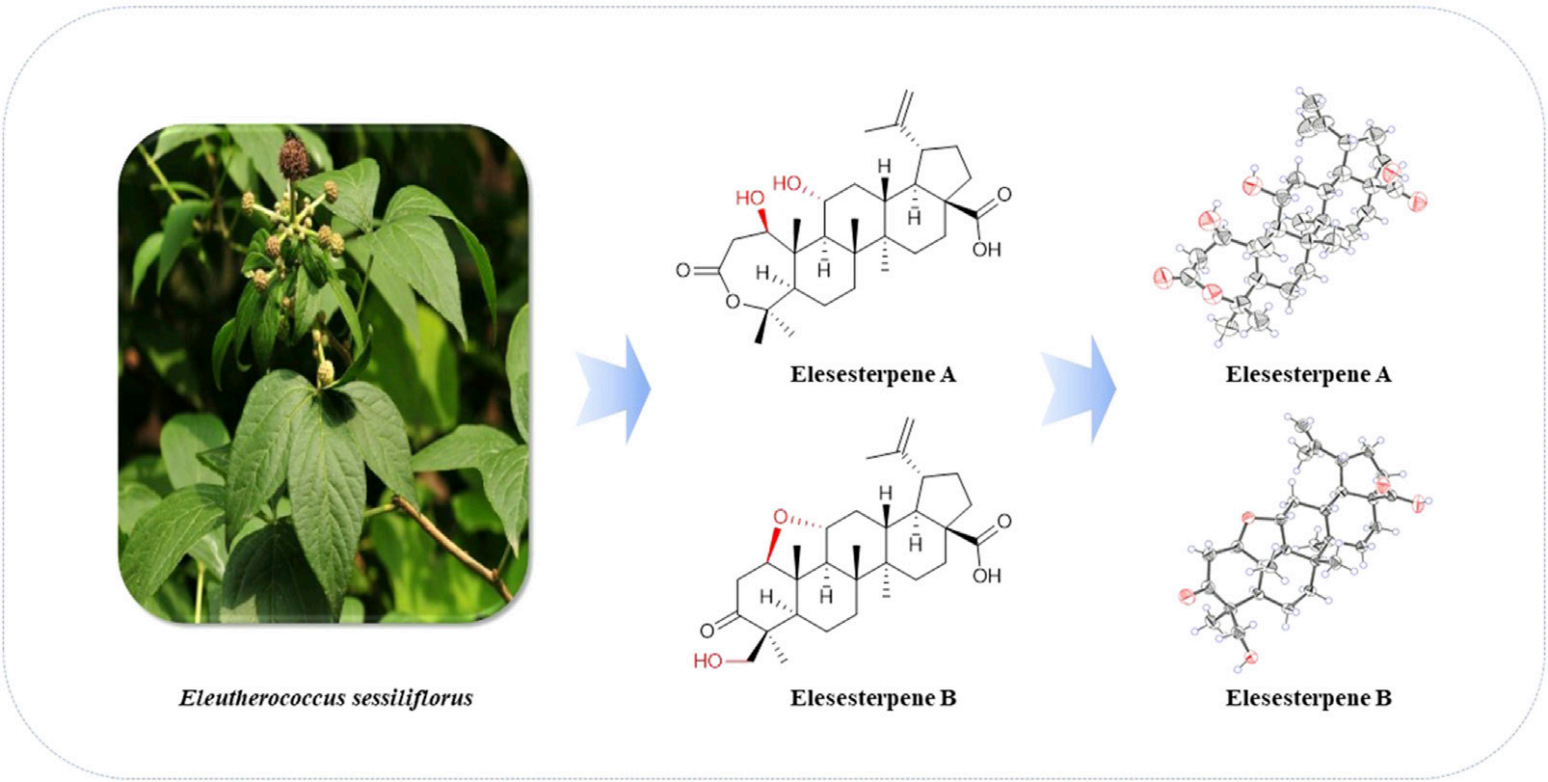
Structures of **1**‐**2** from *Eleutherococcus sessiliflorus*.

## Introduction

Triterpenoids, a kind of structural diversity secondary metabolites in the plant kingdom, are derived from the cyclization of squalene through different pathways and are widely distributed in various plants (Salvador et al., 2012; Thimmappa et al., 2014). Most triterpenoids are tetracyclic or pentacyclic triterpenes; pentacyclic triterpenes can be roughly divided into four subtypes, including oleanane, ursane, lupane, and friedelane.

**FIGURE 1:**
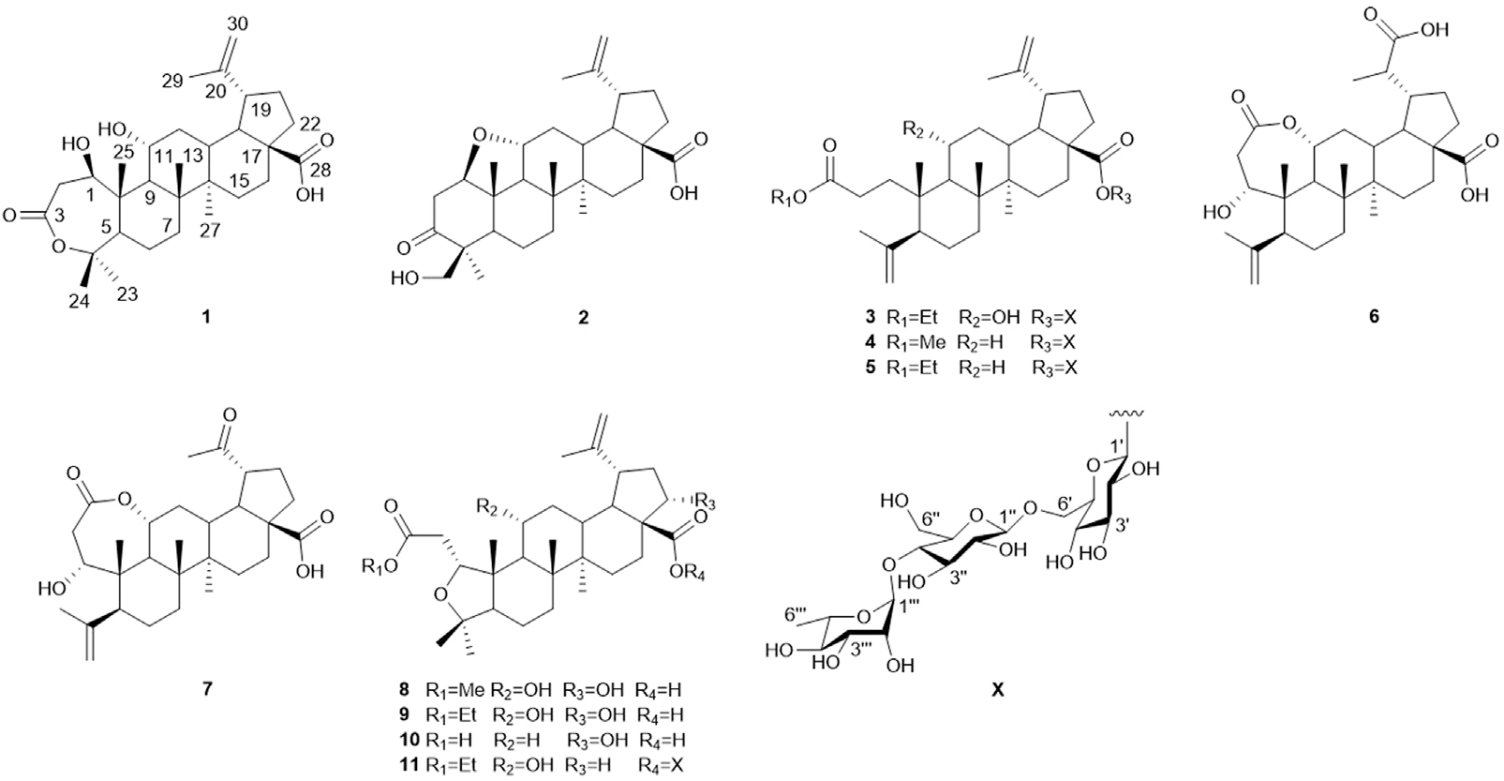
Structures of compounds **1**‐**11**.

*E. sessiliflorus* is widely distributed in Northeast China. It is a type of folk herbal medicine which helps in nourishing liver and kidney, strengthening body and bones, and is used for treating cerebrovascular diseases, tumors, rheumatism, and arthralgia (Jung et al., 2005; Lee et al., 2012). Abundant secondary metabolites, mainly flavonoids and triterpenoids, have been isolated from *E. sessiliflorus* (Yoshizumi et al., 2006; Zhang et al., 2019). Among them, 3,4-seco-lupane–type triterpenoids are characteristic chemical constituents, which exhibit many pharmacological activities, such as antiproliferative, antidepressant, and hepatoprotective activities (Bian et al., 2017; Bian et al., 2018). In the current study, we describe six triterpenoids, four triterpenoid glycosides, and one nortriterpenoid (Figure 1) from the leaves of *E. sessiliflorus*, including their isolation, structural elucidation, the anti-inflammatory activity on BV2 cells, and antiproliferative activities on HepG2, A549, and LN229 cell lines.

## Experimental Section

### General Experimental Procedures

HR-ESI-MS spectra were selected on a Thermo Scientific Orbitrap Fusion™ Lumos™ Tribrid™ mass spectrometer (Thermo, America). GC-MS analysis was performed on an Agilent 7890A system with a DB-1701 (30 m × 0.25 mm, 0.25 μm) capillary column (Agilent Technologies, America). NMR spectra were collected by a Bruker DPX-600 spectrometer (^1^H: 600 MHz, ^13^C: 150 MHz) with the tetramethylsilane as an internal standard and pyridine-*d*_*5*_ (Cambridge Isotope Laboratories Inc., America) as deuterated reagents. The melting point was determined on a melting point apparatus (XPF-550C, CK-300, Shanghai Caikon Optical Instrument Co., Ltd. China). Preparative HPLC was performed with a Shimadzu Shim-pack GIST C18 column (20 × 250 mm, 5 μm), equipped with RID-20A detector, and LC20AR HPLC pump flow rates were 5 ml/min (Shimadzu Corporation, Japan). Optical rotations were measured using a Jasco P-2000 digital polarimeter. X-ray crystallographic data were measured by Bruker D8 Venture. Column chromatography was carried out using silica gel (80–100 mesh and 200–300 mesh, Qingdao Marine Chemical Inc., China) and ODS (50 μm, YMC Company Ltd., Japan). Thin-layer chromatography was conducted with GF254 (Qingdao Marine Chemical Inc., China), which was observed with a UV lamp (254 and 366 nm) and heated after spritzing with 10% H_2_SO_4_ in alcohol, and 10% H_2_SO_4_-EtOH reagent was sprayed and heated at 130°C for 5 min to detect spots.

### Plant Material

The leaves of *E. sessiliflorus* were collected from Dandong, Liaoning Province of China in August 2020, and identified by Prof. Rui-Feng Fan of Heilongjiang University of Chinese Medicine. The voucher specimen (No. 20200821) was deposited at Heilongjiang University of Chinese Medicine.

### Extraction and Isolation

The dried leaves of *E. sessiliflorus* (30 kg) were extracted under reflux with EtOH-H_2_O (2 h × 3 times, 70:30, v/v). The crude extracts were eluted by HP-20 macroporous resin with EtOH-H_2_O (0:100, 40:60, 95:5, v/v), yielding 0% EtOH-H_2_O (1.4 kg), 40% EtOH-H_2_O (1.1 kg), and 95% EtOH-H_2_O (0.4 kg) elution fractions.

The 95% EtOH-H_2_O elution fractions (0.4 kg) were subjected to silica gel column with 200–300 mesh, eluted with the solvent system CH_2_Cl_2_-CH_3_OH (100:0, 80:1, 50:1, 30:1, 20:1, 15:1, 10:1, 5:1, 2:1, 0:100, v/v) yielding nine fractions (Fr. A-Fr. I). Based on TLC analysis, Fr. D (10.0 g) was separated by ODS, and forty-five subfractions were obtained. Fr. D-16 was further separated with preparative HPLC to afford **8** (2.8 mg) and **10** (12.5 mg). Fr. D-18 was purified by preparative HPLC to afford **6** (7.4 mg), **7** (9.8 mg), and **9** (55.7 mg). Fr. D-24 was submitted by preparative HPLC to afford **1** (18.5 mg) and **2** (5.5 mg). Fr. H (59.3 g) was separated by ODS, and forty-two subfractions were obtained. Fr. H-19 was further purified by preparative HPLC to afford **11** (27.4 mg). Fr. H-29 was separated by preparative HPLC to afford **3** (13.6 mg). Fr. H-32 was purified by preparative HPLC to afford **4** (15.4 mg) and **5** (17.3 mg).

### Hydrolysis of Compounds 3, 4, 5, and 11

Each compound (1.0 mg) was hydrolyzed in 2 ml (2 mol/L) of HCL and incubated in water bath at 80°C for 4 h. The mixture was concentrated under vacuum, and the resulting residue was suspended in water and extracted with ethyl acetate (3 × 2.0 ml). And the aqueous layer was evaporated dry under reduced pressure. The residue was dissolved in dry pyridine (1 ml) with L-cysteine methyl ester hydrochloride (2 ml, 0.1 mol/L) and shaken at 60°C for 1 h. Subsequently, N-trimethylsilylimidazole was added and heated at 60°C for 1 h. The reaction mixture was suspended in 1.0 ml H_2_O and extracted with n-hexane (3 × 1.0 ml). The layer of n-hexane was directly analyzed by Agilent GC-MS (7890A) using a DB-1701 column. Temperatures of both injector and detector were 250°C. The split ratio was 10:1. A temperature gradient system was used for the oven, starting at 220°C and increasing up to 270°C at a rate of 5°C/min, and held for 10 min at the final temperature. The absolute configurations of the sugar components were determined by comparison with the retention times of the authentic sugars (D-glucose, 10.72 min; L-rhamnose, 8.78 min).

### Cytotoxic Assay

BV2 microglial cells, HepG2, and LN229 cells were incubated in high-glucose DMEM with 10% heat-inactivated FBS, containing 1% antibiotic (penicillin-streptomycin) at 37°C in 5% CO_2_. Additionally, A549 cells were cultured in F_12_ (10% FBS, 100 IU/ml penicillin, and 100 μg/ml streptomycin) (Ko et al., 2016).

Three human cancer cell lines (HepG2, A549, and LN229) were incubated at 2 × 10^5^ cells/mL in a 96-well microplate for 12 h. Each tumor cell line was exposed to the test compounds at various concentrations (0, 20, 40, 60, 80, and 100 μM) for 24 h. Then the cells were tested with 0.5 mg/ml CCK-8 and incubated for 2 h at 37°C in 5% CO_2_.

BV2 microglial cells were cultured in a 96-well microplate, which was treated with 1 μg/ml LPS for 12 h. Then, they were treated with different concentrations of the compounds **1–11** (0, 20, 40, 60, 80, and 100 μM) for 12 h for the detection of nitric oxide (NO) content in the supernatant of cell culture medium. After the Griess reaction, they were incubated for 0.5 h at 37°C in 5% CO_2_. The absorbance of cells was determined at 490 nm by a microplate reader.

### X-Ray Crystallographic Analysis

Crystallographic data of **1** and **2** were collected on a Bruker D8 venture diffractometer employing Ga Kα radiation. The structures were refined with full-matrix calculations using SHELXL 2018/3 (Sheldrick, 2015). Deposition numbers CCDC 2111722 and 2114935 could be obtained free of charge from the Cambridge Crystallographic Data Centre *via* www.ccdc.cam.ac.uk/data_request/cif.

*Elesesterpene A (* ***1*** *)*: C_30_H_46_O_6_, M = 502.67, T = 173 K, V = 2648.7 (11), D_calcd_ = 1.261 g/cm^3^, Z = 4, tetragonal, p43, *a* = 9.1862 (16) Å, *b* = 9.1862 (16) Å, *c* = 31.388 (7) Å, *α* = *β* = *γ* = 90°, F (000) = 1,096, GOF = 1.041, 4.187° ≤ *θ* ≤ 60.632°, -11 ≤ *h* ≤ 10, -11 ≤ *k* ≤ 11, -33 ≤ *l* ≤ 39, data/restraints/parameters 5370/1/334, final *R* indices *R*_*1*_ = 0.0770 (w*R*_*2*_ = 0.1835) [*I* > 2*σ* (*I*)] for 5370 independent reflections [*R*_int_ = 0.1090], *R* indices (all data) *R*_*1*_ = 0.1561 (w*R*_*2*_ = 0.2241) for reflections were collected. Flack parameter: 0.0 (4). The deposited number CCDC of **1** was 2111722.

*Elesesterpene B (* ***2*** *)*: C_30_H_44_O_5_, C_5_H_5_N, M = 563.75, T = 173 K, V = 1,485.8 (2), D_calcd_ = 1.260 g/cm^3^, Z = 2, monoclinic, p1211, *a* = 13.6515 (10) Å, *b* = 6.7760 (6) Å, *c* = 17.1284 (14) Å, *α* = *γ* = 90°, *β* = 110.325 (4)°, F (000) = 612, GOF = 1.069, 2.393° ≤ *θ* ≤ 60.133°, -15 ≤ *h* ≤ 17, -8 ≤ *k* ≤ 5, -19 ≤ *l* ≤ 21, data/restraints/parameters 5453/1/377, final *R* indices *R*_*1*_ = 0.0427 (w*R*_*2*_ = 0.0970) [*I* > 2*σ* (*I*)] for 5453 independent reflections (*R*_int_ = 0.0476), *R* indices (all data) *R*_*1*_ = 0.0601 (w*R*_*2*_ = 0.1061) for reflections were collected. Flack parameter: 0.0 (2). The deposited number CCDC of **2** was 2114935.

## Results and Discussion

### Structure Elucidation of Compounds

**FIGURE 2:**
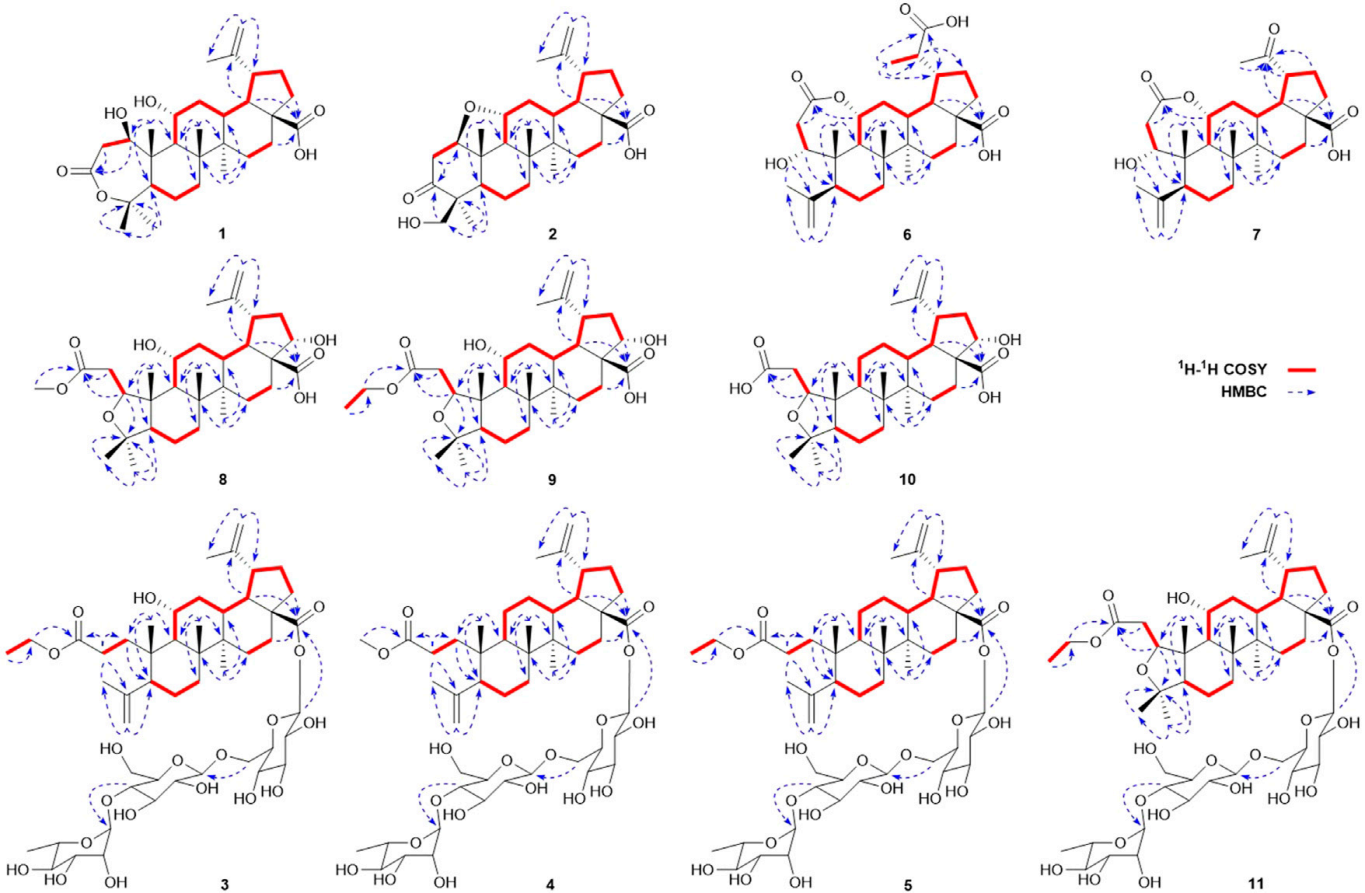
Key ^1^H‐^1^H COSY and HMBC correlations of compounds **1**‐**11**.

**FIGURE 3:**
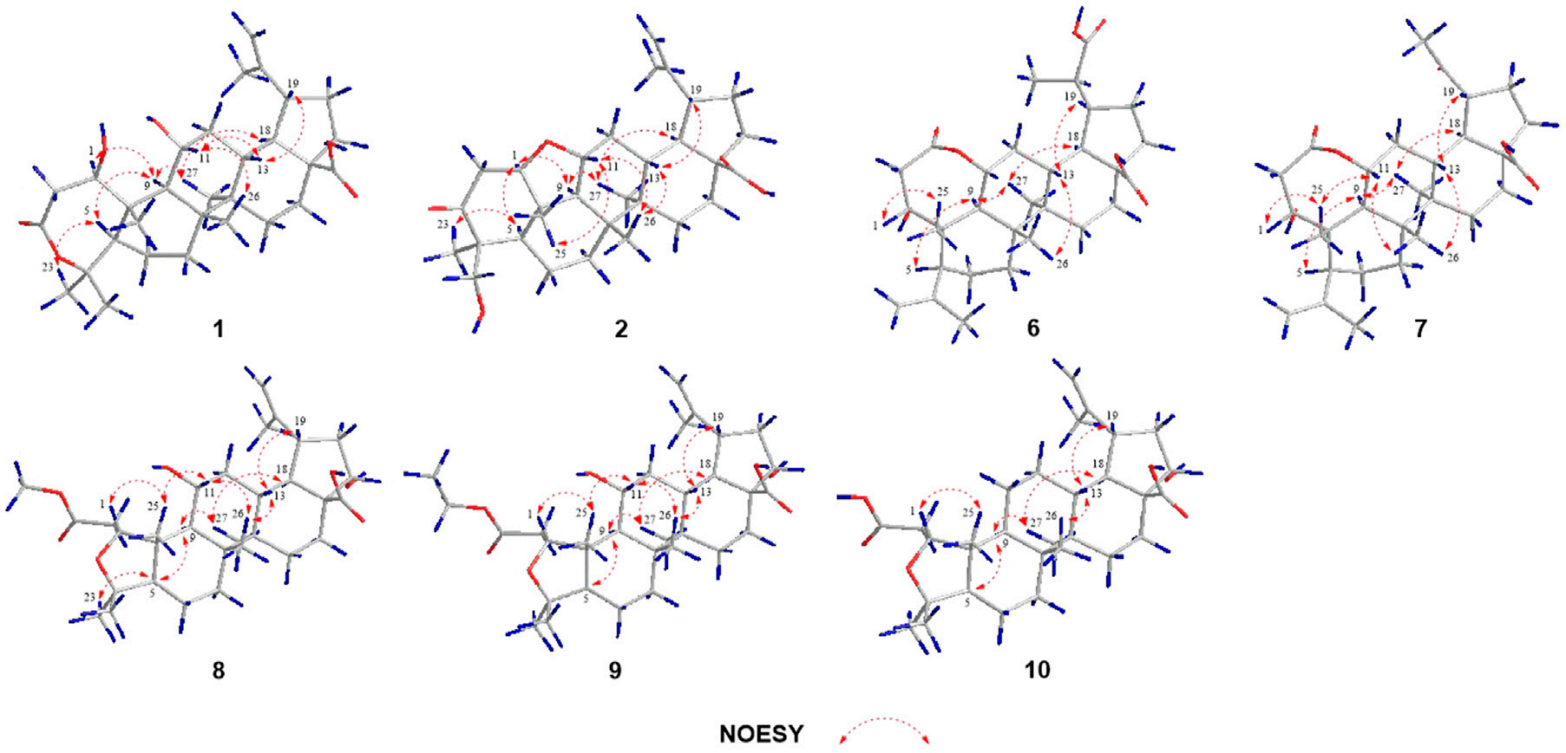
Key NOESY correlations of compounds **1**‐**2**, **6**‐**10**.

**FIGURE 4:**
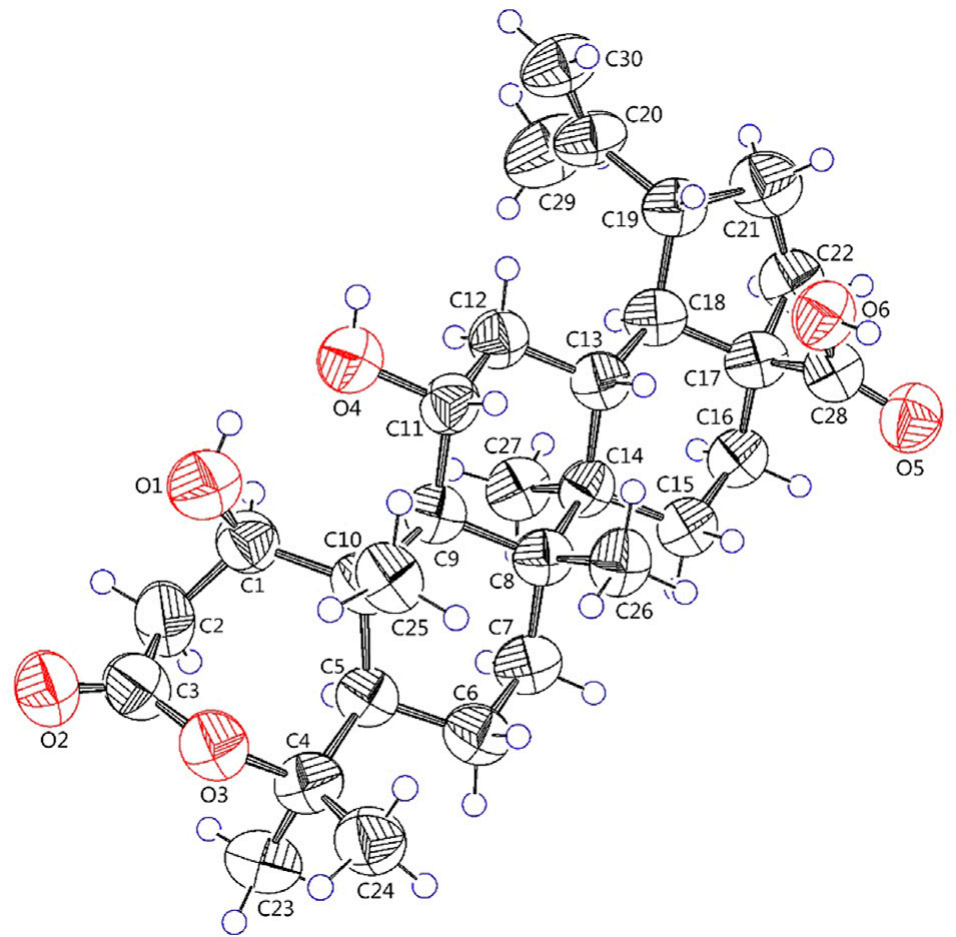
X-ray crystallographic structure of **1**.

Elesesterpene A (**1**) was isolated as a white acicular crystal. It had a molecular formula of C_30_H_46_O_6_, as established by positive high-resolution–electrospray ionization–mass spectrometry (HR-ESI-MS) ion peak at *m/z* 503.3360 ([M + H] ^+^, calculated for C_30_H_47_O_6_, 503.3373). The ^1^H-NMR spectrum of **1** (Table 1) showed the characteristic signals of six methyl signals [*δ*_H_ 1.45 (3H, s, H_3_-23), 1.36 (3H, s, H_3_-24), 1.47 (3H, s, H_3_-25), 1.11 (3H, s, H_3_-26), 1.08 (3H, s, H_3_-27), and 1.74 (3H, s, H_3_-29)] and two terminal alkene protons [*δ*_H_ 4.65 (H, s, H-30a) and 4.86 (H, s, H-30b)]. Combined with the ^13^C-NMR, DEPT, and HSQC spectra analyses, six methyl carbons [*δ*_C_ 29.6 (C-23), 27.8 (C-24), 14.1 (C-25), 17.4 (C-26), 14.4 (C-27), and 19.6 (C-29)], a pair of olefinic carbon [*δ*_C_ 150.7 (C-20) and 110.0 (C-30)], two oxygenated tertiary carbons [*δ*_C_ 79.2 (C-1) and 69.6 (C-11)], two carboxyl carbons [*δ*_C_ 172.9 (C-3) and 178.8 (C-28)] were assigned. The ^1^H-^1^H COSY correlations (Figure 2) showed the main fragment of the triterpenoid framework based on the cross-peaks H-9/H-11/H_2_-12/H-13/H-18/H-19/H_2_-21/H_2_-22. In addition, three other fragments (H-1/H_2_-2; H-5/H_2_-6/H_2_-7; and H_2_-15/H_2_-16) are shown in Figure 2. The HMBC correlations (Figure 2) of H_3_-23, H_3_-24/C-4; H-1, H_2_-2/C-3 combined with the above NMR signals indicated that **1** could be a triterpenoid with 3,4-lactone. The HMBC correlations of H_2_-30/C-19, C-29; H-18/C-20 defined the Δ^20 (30)^ terminal double bond as a part of the C-20 isopropenyl moiety. Thus, the 2D structure of **1** was similar to that of viburolide, which was reported from *Viburnum aboricolum*, except for the presence of two hydroxy groups at C-1 (*δ*_C_ 79.2) and C-11 (*δ*_C_ 69.6) in **1** rather than a hydroxy group at C-6 in viburolide (Ku et al., 2003). The relative configuration of **1** was determined by the analysis of the NOESY experiment (Figure 3). Based on biosynthesis principles, H_3_-26 was tentatively assigned *β*-orientation and H_3_-27 was assigned *α*-orientation as in lupane-type triterpenoids. The correlations of H-1/H-5/H-9/H_3_-27; H-11/H_3_-26 were observed. Therefore, it was inferred that H-1 and H-11 were *α*-orientation and *β*-orientation, respectively. Furthermore, the X-ray crystal structure (Figure 4) of **1** was obtained by crystallization from MeOH at 24°C [Ga Kα radiation, Flack parameter = 0.0 (4), CCDC 2111722], which unambiguously determined the absolute configuration of **1** as 1*R*, 5*R*, 8*R*, 9*S*, 10*R*, 11*R*, 13*R*, 14*R*, 17*S*, 18*R*, 19*R*.

**TABLE 1:** ^1^H (600 MHz) and ^13^C NMR (150 MHz) data in pyridine-*d*_*5*_ for **1**–**2** and **6**–**7**.

No	1		2		6		7	
	*δ*_C_	*δ*_H_ (*J* in Hz)	*δ*_C_	*δ*_H_ (*J* in Hz)	*δ*_C_	*δ*_H_ (*J* in Hz)	*δ*_C_	*δ*_H_ (*J* in Hz)
**1**	79.2	4.35 (br s)	84.9	3.86 (dd, 7.1, 12.5)	70.5	3.76 (d, 8.0)	70.4	3.72 (d, 8.2)
**2**	41.5	2.97 (dd, 3.4, 14.2)	43.9	2.84 (dd, 12.5, 17.5)	38.8	2.89 (dd, 8.0, 14.8)	38.7	2.83 (dd, 8.2, 14.8)
		3.21 (br d, 14.2)		3.01 (dd, 7.1, 17.5)		3.19 (d, 14.8)		3.11 (d, 14.8)
**3**	172.9	—	215.0	—	173.1	—	172.8	—
**4**	84.4	—	53.0	—	147.7	—	147.6	—
**5**	52.0	2.00 (d, 12.1)	52.0	1.44 (m)	49.5	2.92 (m)	49.5	2.91 (dd, 2.8, 13.3)
**6**	24.2	1.29 (overlap)	21.0	1.89 (overlap)	25.1	1.45 (m)	25.1	1.44 (m)
		1.59 (overlap)		1.96 (m)		1.85 (overlap)		1.82 (dd, 3.9, 13.3)
**7**	33.6	1.35 (overlap)	35.2	1.41 (dt, 2.9, 13.9)	32.4	1.20 (overlap)	32.3	1.17 (m)
				1.54 (m)		1.40 (m)		1.38 (m)
**8**	42.3	—	39.9	—	41.5	—	41.6	—
**9**	57.0	1.83 (overlap)	56.4	1.75 (d, 11.0)	43.8	2.82 (d, 9.8)	44.0	2.73 (d, 9.8)
**10**	45.5	—	40.3	—	44.1	—	44.1	—
**11**	69.6	4.03 (m)	77.8	4.03 (td, 4.8, 11.0)	75.5	4.72 (q, 9.8)	75.1	4.58 (q, 9.8)
**12**	37.4	1.65 (overlap)	34.0	1.48 (m)	35.1	2.41 (m)	34.8	1.90 (m)
		2.43 (br d, 12.1)		2.55 (m)		2.49 (m)		1.98 (m)
**13**	37.5	3.02 (t, 12.1)	39.6	2.77 (td, 2.0, 12.3)	35.5	2.94 (m)	34.5	2.70 (ddd, 4.7, 11.1, 13.6)
**14**	42.6	—	42.8	—	42.4	—	42.0	—
**15**	30.0	1.27 (overlap)	31.4	1.22 (m)	29.8	1.13 (m)	29.5	1.13 (m)
		1.69 (overlap)		1.89 (overlap)		1.77 (m)		1.73 (m)
**16**	32.6	1.56 (overlap)	32.2	1.57 (m)	32.6	1.49 (td, 3.3, 13.7)	32.1	1.51 (td, 3.7, 13.1)
		2.62 (d, 12.2)		2.60 (dt, 3.1, 12.8)		2.60 (br d, 13.7)		2.58 (dt, 3.1, 13.1)
**17**	56.5	—	57.0	—	56.8	—	56.3	—
**18**	49.0	1.85 (overlap)	49.5	1.98 (t, 11.0)	49.7	2.25 (t, 10.8)	49.3	2.30 (t, 11.1)
**19**	47.2	3.51 (td, 2.1, 11.1)	47.7	3.46 (td, 4.7, 11.0)	44.0	2.88 (m)	51.9	3.64 (td, 4.6, 11.1)
**20**	150.7	—	151.2	—	42.9	3.06 (m)	211.1	—
**21**	31.1	1.52 (m)	31.0	1.49 (m)	25.4	2.16 (m)	28.8	1.58 (m)
		2.22 (m)		2.21 (m)		2.35 (m)		2.26 (m)
**22**	37.2	1.57 (overlap)	37.3	1.60 (m)	37.1	1.75 (m)	37.0	1.58 (m)
	—	2.27 (m)	—	2.29 (m)	—	2.19 (m)	—	2.18 (m)
**23**	29.6	1.45 (s)	25.2	1.25 (s)	113.8	5.02 (br s)	113.8	5.01 (br s)
						5.12 (br s)		5.11 (br s)
**24**	27.8	1.36 (s)	66.0	3.92 (d, 11.0)	23.5	1.87 (s)	23.5	1.86 (s)
				4.10 (d, 11.0)				
**25**	14.1	1.47 (s)	11.8	1.04 (s)	18.9	1.03 (overlap)	18.9	0.98 (s)
**26**	17.5	1.11 (s)	16.1	1.08 (s)	17.8	1.03 (overlap)	17.7	0.97 (s)
**27**	14.4	1.08 (s)	15.8	1.13 (s)	13.8	1.18 (s)	13.6	1.07 (s)
**28**	178.8	—	178.7	—	178.9	—	178.5	—
**29**	19.6	1.74 (s)	19.7	1.76 (s)	18.1	1.32 (d, 7.0)	29.2	2.12 (s)
**30**	110.0	4.65 (s)	109.8	4.73 (br s)	178.0	—	—	—
		4.86 (s)		4.90 (br d, 1.4)				

**FIGURE 5:**
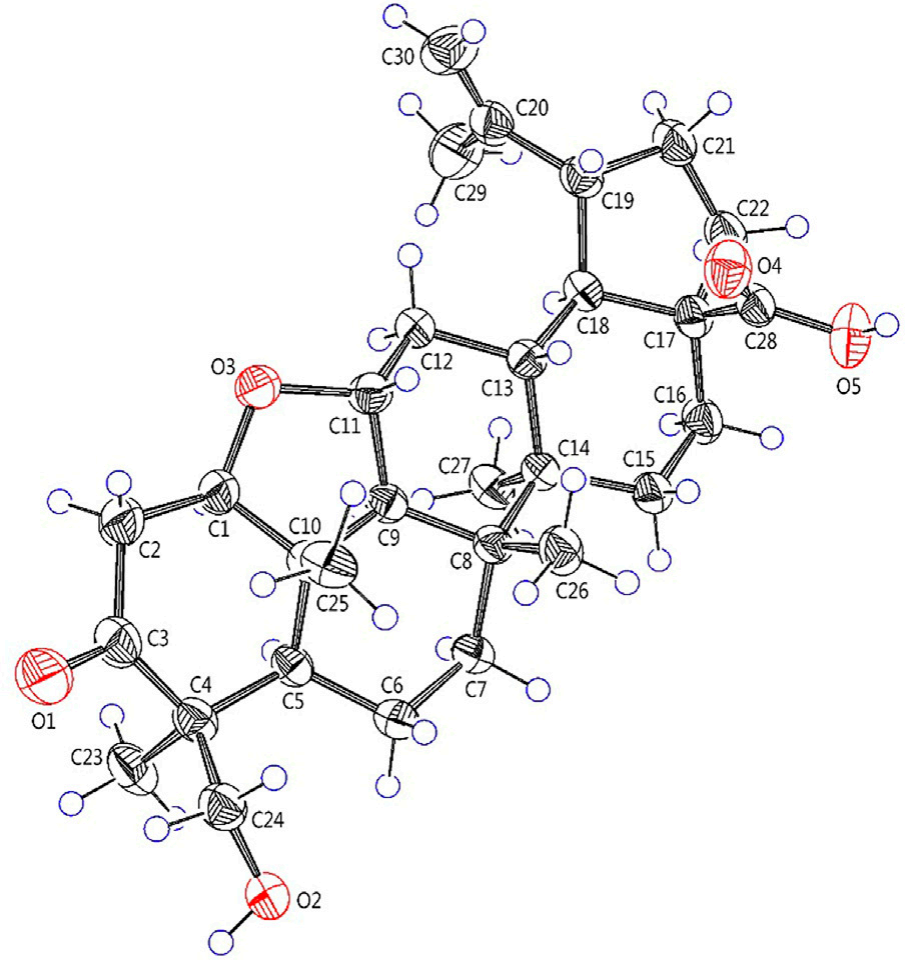
X-ray crystallographic structure of **2**.

Elesesterpene B (**2**) was obtained as a white acicular crystal. HR-ESI-MS showed a molecular ion at *m/z* 485.3260 ([M + H] ^+^, calculated for C_30_H_45_O_5_, 485.3267), which agreed with the molecular formula (C_30_H_44_O_5_). Based on the analysis of the NMR data of **2** (Table 1), five methyl carbons, one carbonyl carbon, one carboxyl carbon, a set of double bonds, one oxygenated secondary carbon, and two oxygenated tertiary carbons could be inferred. The ^1^H-^1^H COSY correlations of **2** revealed the existence of four fragments identical with that of **1** (Figure 2). The six-membered ring A was distinct from **1**, which was determined by the key HMBC correlations of H_2_-2/C-1, C-3; H_3_-23/C-4, C-5, and C-24; H_2_-24/C-3 (Figure 2). Therefore, the planar structure of **2** was established as shown in Figure 1. The absence of correlation between C-1 and C-11 in HMBC made it impossible to judge that C-1 and C-11 were connected by an oxygen bridge, and this connection was proved further by single-crystal X-ray diffraction experiments. The NOESY correlations (Figure 3) of H-1/H-5/H-9/H_3_-27; H-11/H_3_-25/H_3_-26 indicated that the relative configuration of **2** was similar to that of **1**. A crystal of **2** was acquired from MeOH at 24°C, and a single-crystal X-ray diffraction experiment (Figure 5) was conducted using Ga Kα radiation [Flack parameter = 0.0 (2), CCDC 2114935] to confirm the absolute configuration of **2** as 1*R*, 4*S*, 5*R*, 8*R*, 9*S*, 10*S*, 11*R*, 13*R*, 14*R*, 17*S*, 18*R*, 19*R*.

**FIGURE 6:**
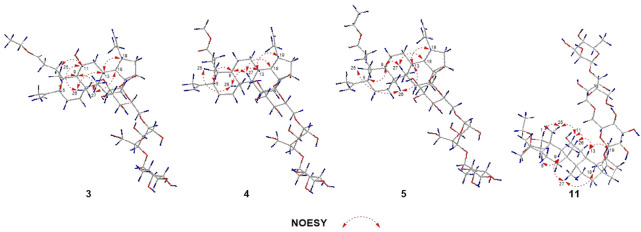
Key NOESY correlations of compounds **3**‐**5**, **11**.

Elesesterpene C (**3**), a yellow amorphous solid, has a molecular formula of C_50_H_80_O_19_ as deduced from HR-ESI-MS ion at *m/z* 985.5359 ([M + H] ^+^, calculated for C_50_H_81_O_19_, 985.5372). The ^1^H and ^13^C-NMR data (Table 2) of **3** were similar to an inermoside obtained previously from *Acanthopanax senticosus*, and the only difference between the two compounds was that the methyl ester at C-3 was replaced by an ethyl ester which was supported by a typical ethyl characteristic carbon signature (*δ*_C_ 59.8 and 14.3) (Park et al., 2000). According to the ^1^H-^1^H COSY correlation of H_2_-1′′′′/H_3_-2′′′′ and the HMBC correlation of H_2_-1′′′′/C-3 (Figure 2), the planar structure of **3** was determined and established as shown in Figure 1. The NOESY correlations (Figure 6) of H-5/H-9/H_3_-27 and H-11/H_3_-25/H_3_-26 indicated H-5 and H-11 were of *α-* and *β*-orientation, respectively.

**TABLE 2:** ^1^H (600 MHz) and ^13^C NMR (150 MHz) data in pyridine-*d*_*5*_ for **3**–**5**.

No	3		4		5	
	*δ*_C_	*δ*_H_ (*J* in Hz)	*δ*_C_	*δ*_H_ (*J* in Hz)	*δ*_C_	*δ*_H_ (*J* in Hz)
**1**	37.4	1.96 (overlap)	34.6	1.67 (overlap)	34.6	1.71 (overlap)
		3.04 (td, 5.9, 13.1)				
**2**	30.2	2.59 (m)	28.5	2.28 (m)	28.7	2.30 (m)
		3.13 (m)		2.48 (m)		2.48 (m)
**3**	174.9	—	174.3	—	173.9	—
**4**	148.3	—	147.9	—	147.9	—
**5**	51.2	2.16 (m)	50.2	2.00 (dd, 2.6, 12.5)	50.1	2.01 (dd, 2.3, 12.8)
**6**	25.1	1.31 (m)	24.8	1.27 (m)	24.8	1.28 (m)
		1.76 (m)		1.70 (overlap)		1.71 (overlap)
**7**	33.7	1.23 (overlap)	32.7	1.21 (overlap)	32.8	1.22 (overlap)
		1.36 (m)		1.32 (overlap)		1.33 (overlap)
**8**	42.3	—	40.7	—	40.7	—
**9**	45.7	1.92 (d, 10.7)	40.9	1.55 (m)	40.9	1.56 (m)
**10**	40.0	—	39.3	—	39.4	—
**11**	69.6	4.15 (m)	21.5	1.17 (overlap)	21.6	1.17 (overlap)
				1.30 (overlap)		1.32 (overlap)
**12**	37.8	1.61 (m)	25.8	1.18 (overlap)	25.8	1.17 (overlap)
		2.33 (dt, 4.1, 12.8)		1.84 (m)		1.84 (m)
**13**	37.2	2.87 (ddd, 3.5, 10.3, 12.8)	38.2	2.67 (td, 2.8, 11.8)	38.3	2.67 (td, 2.8, 11.6)
**14**	42.9	—	43.1	—	43.1	—
**15**	30.1	1.23 (overlap)	30.1	1.20 (overlap)	30.1	1.20 (overlap)
		1.90 (m)		1.97 (td, 3.3, 14.0)		1.97 (td, 3.0, 13.5)
**16**	32.1	1.52 (td, 3.2, 13.0)	32.1	1.50 (overlap)	32.1	1.50 (overlap)
		2.64 (dt, 3.4, 13.0)		2.63 (dt, 3.3, 13.0)		2.63 (dt, 3.0, 12.9)
**17**	56.9	—	56.9	—	56.9	—
**18**	49.3	1.80 (overlap)	49.6	1.75 (overlap)	49.6	1.74 (overlap)
**19**	47.1	3.35 (td, 5.0, 11.0)	47.3	3.37 (td, 5.0, 10.9)	47.3	3.37 (td, 5.0, 10.9)
**20**	150.3	—	150.8	—	150.7	—
**21**	30.8	1.40 (m)	30.8	1.42 (m)	30.8	1.42 (m)
		2.11 (m)		2.15 (m)		2.15 (m)
**22**	36.6	1.47 (m)	36.7	1.49 (m)	36.7	1.48 (m)
		2.20 (m)		2.21 (m)		2.20 (m)
**23**	113.9	4.88 (br s)	113.6	4.77 (br s)	113.7	4.78 (br s)
		4.96 (br s)		4.90 (br s)		4.91 (br s)
**24**	23.7	1.80 (s)	23.4	1.71 (s)	23.4	1.72 (s)
**25**	20.8	1.19 (s)	20.3	0.77 (s)	20.3	0.78 (s)
**26**	17.3	1.20 (s)	16.2	1.13 (s)	16.2	1.14 (s)
**27**	14.7	1.11 (s)	14.7	1.03 (s)	14.7	1.03 (s)
**28**	174.8	—	174.9	—	174.9	—
**29**	19.4	1.66 (s)	19.4	1.73 (s)	19.4	1.71 (s)
**30**	110.2	4.60 (br s)	110.0	4.73 (br s)	110.0	4.72 (br s)
		4.79 (br s)		4.88 (br s)		4.87 (br s)
**1′**	95.2	6.32 (d, 8.2)	95.2	6.35 (d, 8.2)	95.2	6.34 (d, 8.2)
**2′**	74.0	4.09 (m)	74.0	4.10 (overlap)	74.0	4.09 (overlap)
**3′**	78.6	4.21 (t, 9.4)	78.6	4.22 (t, 9.0)	78.6	4.22 (t, 9.0)
**4′**	70.8	4.30 (t, 9.4)	70.8	4.32 (t, 9.0)	70.8	4.31 (t, 9.0)
**5′**	77.9	4.09 (m)	77.9	4.10 (m)	77.9	4.10 (m)
**6′**	69.3	4.27 (dd, 4.9, 11.6)	69.3	4.28 (dd, 4.7, 11.3)	69.3	4.28 (dd, 4.7, 11.3)
		4.68 (overlap)		4.68 (overlap)		4.68 (overlap)
**1″**	105.0	4.94 (d, 7.7)	105.1	4.93 (d, 8.6)	105.0	4.93 (d, 8.6)
**2″**	75.2	3.92 (t, 7.7)	75.2	3.93 (t, 8.6)	75.2	3.93 (t, 8.6)
**3″**	76.4	4.12 (m)	76.4	4.13 (t, 9.4)	76.4	4.13 (m)
**4″**	78.1	4.40 (t, 9.3)	78.1	4.40 (t, 9.4)	78.1	4.40 (t, 9.3)
**5″**	77.1	3.64 (dt, 3.1, 9.3)	77.1	3.64 (overlap)	77.1	3.64 (m)
**6″**	61.2	4.07 (m)	61.2	4.08 (dd, 3.2, 10.4)	61.2	4.08 (m)
		4.19 (m)		4.19 (br d, 10.4)		4.19 (m)
**1‴**	102.6	5.85 (br s)	102.6	5.85 (br s)	102.6	5.85 (br s)
**2‴**	72.5	4.67 (overlap)	72.5	4.67 (overlap)	72.5	4.67 (overlap)
**3‴**	72.7	4.54 (dd, 3.3, 9.2)	72.7	4.54 (dd, 3.3, 9.2)	72.7	4.54 (dd, 3.2, 9.2)
**4‴**	73.9	4.33 (m)	73.9	4.33 (t, 9.2)	73.9	4.33 (t, 9.2)
**5‴**	70.2	4.93 (overlap)	70.2	4.95 (m)	70.2	4.95 (m)
**6‴**	18.4	1.69 (d, 6.2)	18.4	1.69 (d, 6.2)	18.4	1.69 (d, 6.2)
**1′′′′**	59.8	4.09 (m)	51.3	3.65 (s)	60.3	4.15 (m)
**2′′′′**	14.3	1.08 (t, 7.1)	—	—	14.3	1.15 (t, 7.1)

Elesesterpene D (**4**), a yellow amorphous solid, has a molecular formula of C_49_H_78_O_18_ as established by the HR-ESI-MS ion at *m/z* 972.5525 ([M + NH_4_] ^+^, calculated for C_49_H_82_NO_18_, 972.5532). It was discovered to be almost consistent with sessiloside isolated from *Acanthopanax sessiliflorus* by comparing the ^1^H and ^13^C NMR data of **4** (Yoshizumi et al., 2006). The HMBC correlation (Figure 2) of H_3_-1′′′′/C-3 showed that the extra methyl ester (*δ*_C_ 51.3, *δ*_H_ 3.65) was connected to C-3 in **4**. The relative configuration of **4** was assigned by the NOESY experiment to be the same as those of **3** (Figure 6).

Elesesterpene E (**5**) was also purified as a yellow amorphous solid. Its molecular formula was determined to be C_50_H_80_O_18_ by the HR-ESI-MS ion at *m/z* 986.5679 [(M + NH_4_) ^+^, calculated for C_50_H_84_NO_18_, 986.5688]. Through comparison of the ^1^H and ^13^C NMR data between **5** and **4**, it was obvious that the methyl ester at C-3 was replaced by an ethyl ester (*δ*_C_ 60.3 and 14.3); this judgment was confirmed by several correlations of H_2_-1′′′′/H_3_-2′′′′; H_2_-1′′′′/C-3 in the ^1^H-^1^H COSY and HMBC (Figure 2). The relative configuration of **5** was entirely consistent with that of **4** (Figure 6).

Elesesterpene F (**6**) was obtained as a colorless amorphous solid with a molecular formula of C_30_H_44_O_7_ based on the HR-ESI-MS ion at *m/z* 517.3179 ([M + H] ^+^, calculated for C_30_H_45_O_7_, 517.3165). The ^1^H-NMR and ^13^C-NMR data of **6** were similar to those of chiisanogenin obtained previously from *Acanthopanax divaricatus*, and the only change was that the Δ^20 (30)^ double bond was replaced by a carboxyl group (Lee et al., 2003). It was supported by correlations of H-20, H_3_-29/C-30 in HMBC (Figure 2). NOESY correlations (Figure 3) between H-1/H_3_-25; H-5/H-9/H_3_-27 were observed. Therefore, H-1 and H-5 were inferred as *β-* and *α*-orientation, respectively.

Elesesterpene G (**7**), a yellow amorphous solid, was determined to be C_29_H_42_O_6_ by HR-ESI-MS at *m/z* 487.3074 ([M + H] ^+^, calculated for 487.3060). Comparison of the ^1^H and ^13^C NMR data (Table 1) of **7** with the data of **6** showed the close similarity between the structures, except for the existence of a ketone carbonyl signal at *δ*_C_ 211.1. The ^13^C NMR and DEPT spectra of **7** resolved 29 carbon signals; these suggested that **7** was a nortriterpenoid. Further analysis of the correlations (H-18, H_3_-29/C-20) established that the ketone carbonyl was straightforwardly assigned to C-19. The relative configuration of **7** was entirely consistent with that of **6** (Figure 3).

Elesesterpene H (**8**) was obtained as a yellow amorphous solid. The molecular formula of the compound was C_31_H_48_O_7_ as established by HR-ESI-MS data [(M + H) ^+^, 533.3467; calculated for C_31_H_49_O_7_, 533.3478]. The ^1^H-NMR and ^13^C-NMR data (Table 3) of **8** were similar to those of acanthosessilioside C obtained from the fruits of *Acanthopanax sessiliflorus*, and it was found to lack a sugar moiety at the C-28 position by contrast (Lee et al., 2012). The correlation of H-1/C-4 in the HMBC (Figure 2) suggested that C-1 and C-4 were connected by an oxygen bridge. Furthermore, the coupling constant between H-22 and H_2_-21 was 5.3 Hz, indicating that H-22 is *β*-orientation (Shirasuna et al., 1997). The NOESY correlations of H-1/H-11/H_3_-25 (Figure 3) indicated that H-1 and H-11 were designated to be of *β*-orientation.

**TABLE 3:** ^1^H (600 MHz) and ^13^C NMR (150 MHz) data in pyridine-*d*_*5*_ for **8**–**11**.

No	8		9		10		11	
	*δ*_C_	*δ*_H_ (*J* in Hz)	*δ*_C_	*δ*_H_ (*J* in Hz)	*δ*_C_	*δ*_H_ (*J* in Hz)	*δ*_C_	*δ*_H_ (*J* in Hz)
**1**	87.2	4.90 (dd, 2.6, 11.5)	87.2	4.88 (dd, 2.5, 11.3)	85.5	4.48 (dd, 3.5, 11.0)	87.1	4.87 (dd, 2.6, 11.4)
**2**	38.5	2.70 (overlap)	38.6	2.65 (overlap)	38.3	2.69 (dd, 3.5, 13.7)	38.6	2.64 (overlap)
		3.70 (dd, 2.6, 13.6)		3.66 (dd, 2.5, 13.6)		2.80 (dd, 11.0, 13.7)		3.65 (overlap)
**3**	173.4	—	172.9	—	174.5	—	172.9	—
**4**	79.2	—	79.1	—	81.0	—	79.1	—
**5**	56.0	1.75 (m)	55.9	1.73 (m)	56.1	1.73 (m)	55.9	1.72 (m)
**6**	18.7	1.39 (overlap)	18.7	1.40 (overlap)	18.8	1.39 (overlap)	18.6	1.36 (m)
		1.44 (m)		1.43 (m)		1.43 (overlap)		1.42 (overlap)
**7**	35.6	1.41 (overlap)	35.5	1.39 (overlap)	34.6	1.41 (overlap)	35.3	1.31 (overlap)
		1.49 (m)		1.48 (m)		1.45 (overlap)		1.42 (overlap)
**8**	42.9	—	42.8	—	41.6	—	42.6	—
**9**	48.9	2.00 (d, 10.7)	48.9	1.97 (d, 11.1)	42.7	1.89 (dd, 2.4, 12.7)	48.7	1.91 (d, 10.9)
**10**	46.9	—	46.9	—	47.8	—	46.8	—
**11**	67.7	4.21 (td, 4.9, 10.7)	67.7	4.19 (m)	23.9	1.22 (d, 12.7)	67.5	4.15 (m)
						1.55 (ddd, 4.4, 13.1, 26.1)		
**12**	37.0	1.67 (dt, 11.6, 13.4)	36.9	1.63 (dt, 11.3, 13.3)	25.6	1.42 (overlap)	36.8	1.50 (overlap)
		2.59 (dt, 4.9, 11.6)		2.56 (dt, 4.1, 11.3)		2.04 (overlap)		2.36 (dt, 4.3, 12.4)
**13**	37.4	3.12 (td, 3.3, 13.4)	37.4	3.08 (td, 3.3, 13.3)	38.6	2.96 (td, 3.4, 12.5)	37.4	2.81 (td, 3.2, 12.4)
**14**	42.7	—	42.6	—	43.3	—	42.7	—
**15**	29.9	1.37 (dt, 3.1, 13.4)	29.9	1.35 (overlap)	30.0	1.34 (dt, 3.2, 13.7)	30.3	1.21 (dt, 3.0, 13.9)
		1.95 (td, 3.8, 13.4)		1.91 (td, 3.4, 13.2)		2.00 (td, 3.6, 13.7)		2.02 (td, 3.0, 13.4)
**16**	27.0	2.48 (td, 3.8, 13.1)	27.0	2.45 (td, 3.4, 13.3)	27.1	2.46 (td, 3.6, 13.3)	32.1	1.55 (td, 3.5, 13.4)
		2.57 (dt, 3.1, 13.1)		2.54 (dt, 2.9, 13.3)		2.56 (dt, 3.2, 13.3)		2.66 (overlap)
**17**	62.7	—	62.7	—	62.7	—	56.9	—
**18**	43.9	2.68 (dd, 3.3, 11.3)	43.8	2.65 (d, 11.2)	44.1	2.61 (t, 11.0)	49.4	1.80 (t, 11.3)
**19**	47.7	3.65 (td, 4.9, 11.3)	47.6	3.61 (td, 4.9, 11.2)	47.9	3.66 (td, 4.9, 11.0)	47.1	3.34 (td, 4.9, 11.3)
**20**	151.4	—	151.3	—	151.8	—	150.4	—
**21**	42.1	1.80 (dd, 4.9, 14.5)	42.0	1.77 (dd, 4.9, 14.5)	42.0	1.81 (dd, 4.9, 14.5)	30.8	1.41 (overlap)
		2.74 (ddd, 5.3, 11.3, 14.5)		2.70 (ddd, 5.3, 11.3, 14.5)		2.74 (ddd, 5.3, 11.3, 14.5)		2.12 (m)
**22**	75.4	4.82 (br d, 5.3)	75.3	4.78 (d, 5.3)	75.5	4.81 (br d, 5.3)	36.6	1.51 (overlap)
								2.21 (dd, 8.2, 12.0)
**23**	24.8	1.16 (s)	24.8	1.15 (s)	24.7	1.14 (s)	24.8	1.15 (s)
**24**	32.5	1.39 (s)	32.5	1.38 (s)	32.7	1.41 (s)	32.5	1.38 (s)
**25**	19.2	1.34 (s)	19.1	1.32 (s)	19.3	1.08 (s)	19.0	1.31 (s)
**26**	17.9	1.15 (s)	17.8	1.12 (s)	17.0	1.10 (s)	17.8	1.16 (s)
**27**	15.1	1.31 (s)	15.1	1.29 (s)	14.9	1.26 (s)	15.1	1.13 (s)
**28**	178.6	—	178.5	—	178.6	—	174.8	—
**29**	19.3	2.02 (s)	19.2	2.00 (s)	19.2	2.05 (s)	19.4	1.68 (s)
**30**	110.5	4.73 (br s)	110.4	4.71 (br s)	110.4	4.83 (br s)	110.1	4.63 (br s)
		4.99 (d, 2.2)		4.97 (d, 1.9)		5.08 (d, 2.1)		4.80 (br s)
**1′**	—	—	—	—	—	—	95.2	6.32 (d, 8.2)
**2′**	—	—	—	—	—	—	74.0	4.10 (m)
**3′**	—	—	—	—	—	—	78.5	4.23 (t, 8.9)
**4′**	—	—	—	—	—	—	70.7	4.31 (t, 8.9)
**5′**	—	—	—	—	—	—	77.9	4.10 (m)
**6′**	—	—	—	—	—	—	69.3	4.28 (dd, 4.9, 11.3)
								4.68 (overlap)
**1″**	—	—	—	—	—	—	104.9	4.94 (d, 8.5)
**2″**	—	—	—	—	—	—	75.2	3.92 (t, 8.5)
**3″**	—	—	—	—	—	—	76.3	4.12 (m)
**4″**	—	—	—	—	—	—	78.1	4.40 (t, 9.4)
**5″**	—	—	—	—	—	—	77.0	3.63 (overlap)
**6″**	—	—	—	—	—	—	61.2	4.07 (overlap)
								4.19 (overlap)
**1‴**	—	—	—	—	—	—	102.6	5.84 (br s)
**2‴**	—	—	—	—	—	—	72.4	4.66 (overlap)
**3‴**	—	—	—	—	—	—	72.6	4.53 (dd, 3.3, 9.2)
**4‴**	—	—	—	—	—	—	73.8	4.33 (t, 9.4)
**5‴**	—	—	—	—	—	—	70.2	4.93 (m)
**6‴**	—	—	—	—	—	—	18.4	1.69 (d, 6.3)
**1′′′′**	51.0	3.62 (s)	59.8	4.11 (m)	—	—	59.8	4.14 (m)
				4.16 (m)				4.19 (m)
**2′′′′**	—	—	14.3	1.12 (t, 7.1)	—	—	14.3	1.11 (t, 7.1)

Elesesterpene I (**9**) was obtained as a yellow amorphous solid with a molecular formula of C_32_H_50_O_7_ as determined by the HR-ESI-MS [(M + H) ^+^, 547.3627; calculated for C_32_H_51_O_7_, 547.3635]. The ^1^H and ^13^C-NMR data of **9** (Table 3) were closely related to those of **8**. Through the ^1^H-^1^H COSY correlation of H_2_-1′′′′/H_3_-2′′′′ and the HMBC correlation of H_2_-1′′′′/C-3, it was determined that C-3 was connected to ethyl ester rather than methyl ester. The relative configurations of **9** were established to be the same as those of **8** by the NOESY experiment (Figure 3).

Elesesterpene J (**10**) was isolated as a white amorphous powder. Its molecular formula was determined as C_30_H_46_O_6_ on the basis of HR-ESI-MS data [(M + H) ^+^, 503.3366; calculated for C_30_H_47_O_6_, 503.3373]. Comparison of the NMR data (Tables 3) of **10** with the data of **8** showed the absence of a methoxy signal and a hydroxyl signal. The correctness of the inference was confirmed by the correlation signals of H-9/H_2_-11/H_2_-12 in ^1^H-^1^H COSY spectra (Figure 2). As with compound **8**, H-1 and H-22 were defined as *β*-orientation.

Elesesterpene K (**11**) was isolated as a yellow amorphous solid. The molecular formula of **11** was established to be C_50_H_80_O_20_ on the basis of its HR-ESI-MS data at *m/z* 1,001.5293 [(M + H) ^+^, calculated for C_50_H_81_O_20_, 1,001.5321]. The ^1^H and ^13^C-NMR data (Tables 3) were similar to those for **9**, with the main difference being the absence of a hydroxyl group and the presence of three sugar units. Acid hydrolysis of **11** released D-glucose and L-rhamnose, which were identified by GC analysis after derivatization. The sugar moieties (*δ*_C_ 95.2, 74.0, 78.5, 70.7, 77.9, 69.3; *δ*_C_ 104.9, 75.2, 76.3, 78.1, 77.0, 61.2; *δ*_C_ 102.6, 72.4, 72.6, 73.8, 70.2, 18.4) were assigned *via* comparison of the experimental and reported NMR data. The correlations of H-18, H_2_-22/C-28 were observed from the HMBC spectrum. The correlation peaks of H-1/H-11/H_3_-25 in the NOESY spectrum showed that H-1 and H-11 have uniform *β-*orientation in space.

### Bioactive Activity

Finally, the antiproliferative activities of these compounds were evaluated against HepG2, A-549, and LN229. And most of the compounds showed significant inhibitory effects. Further analysis of the data showed that compounds **5**, **7**, **8**, and **10** exhibited more extensive and potent effects with the IC_50_ values ranging from 1.05 to 8.60 μM (Table 4).

**TABLE 4:** Antiproliferative bioassays and inhibitory activity against NO production in LPS-stimulated BV2 of compounds **1**–**11**.

Compounds	IC_50_ (μM)^a^	IC_50_ (μM)^a^	IC_50_ (μM)^a^	IC_50_ (μM)^b^
	HepG2	A549	LN229	BV2
**1**	>50	5.36 ± 0.57	5.42 ± 0.59	>50
**2**	16.78 ± 2.05	>50	6.42 ± 0.78	>50
**3**	>50	46.84 ± 4.25	9.04 ± 1.08	2.33 ± 0.31
**4**	43.63 ± 4.02	1.92 ± 0.22	3.01 ± 0.35	25.13 ± 2.61
**5**	8.37 ± 0.98	8.60 ± 0.85	6.84 ± 0.75	15.88 ± 1.35
**6**	0.12 ± 0.02	>50	>50	>50
**7**	5.09 ± 0.89	1.41 ± 0.55	1.05 ± 0.15	21.54 ± 1.95
**8**	5.25 ± 0.95	6.52 ± 0.79	3.63 ± 0.34	>50
**9**	11.56 ± 1.29	10.45 ± 1.26	42.68 ± 3.58	>50
**10**	5.12 ± 0.68	6.97 ± 0.77	7.97 ± 0.82	34.67 ± 3.29
**11**	6.86 ± 0.71	9.89 ± 0.84	>50	30.14 ± 2.89

a IC_50_ was defined as the concentration that resulted in a 50% decrease in cell number.

b IC_50_ was the half-maximal inhibitory concentration of NO production.

Value present means ± SD of triplicate experiments.

The IC_50_ > 50 μM in biological activity was deemed inactive.

The bioactivities of isolated metabolites were also evaluated for the anti-inflammatory assay *in vitro*. All of them exhibited different degrees of suppression on NO production in LPS-activated BV2 microglial cells (Table 4), and compound **3** was the best (IC_50_ = 2.33 ± 0.31 μM).

## Conclusion

*E. sessiliflorus* was a kind of medicinal and edible herbal medicine. Numerous new compounds were isolated from the leaves in our study, including ten triterpenoids (**1–6** and **8–11**) and one nortriterpenoid (**7**). These were worth mentioning; the C-30 of compounds **6** and **7** had a rare change from the double bond to carboxyl and ketone carbonyl groups, respectively; in compound **2**, C-1 and C-11 were linked to form a rare five-membered oxygen ring. All of these changes were reported for the first time. In addition, compounds **3**, **5**, **9**, and **11** all had an extra segment (-OCH_2_CH_3_) in C-3 compared with known triterpenoids. Thus, **3**, **5**, **9**, and **11** were possible to be the artifacts. A possibility was proposed: some esterification reactions occurred during the refluxing extraction of ethanol. To further avoid the introduction of 3-OCH_2_CH_3_, a lot of methods are currently being carried out in our studies, and the results will be reported in due course.

## Data Availability

The datasets presented in this study can be found in online repositories. The names of the repository/repositories and accession number(s) can be found in the article/Supplementary Material.
