# Supplementary material for: Elesesterpenes A–K: Lupane-type Triterpenoids From the Leaves of Eleutherococcus sessiliflorus
Source: Front Chem. 2022 Jan 24;9:813764. doi: 10.3389/fchem.2021.813764 (PMC8819545; doi:10.3389/fchem.2021.813764)

Supplementary Material

**Supplementary Data**

**Supplementary Figures**

**Figure S1**. ^1^H NMR spectrum of Elesesterpene A (**1**) (600 MHz, pyridine‑*d_5_*).

**Figure S2**. ^13^C NMR spectrum of Elesesterpene A (**1**) (150 MHz, pyridine‑*d_5_*).

**Figure S3**. DEPT spectrum of Elesesterpene A (**1**).

**Figure S4**. HSQC spectrum of Elesesterpene A (**1**).

**Figure S5**. HMBC spectrum of Elesesterpene A (**1**).

**Figure S6**. ^1^H-^1^H COSY spectrum of Elesesterpene A (**1**).

**Figure S7**. NOESY spectrum of Elesesterpene A (**1**).

**Figure S8**. HR-ESI-MS spectrum of Elesesterpene A (**1**).

**Figure S9**. ^1^H NMR spectrum of Elesesterpene B (**2**) (600 MHz, pyridine‑*d_5_*).

**Figure S10**. ^13^C NMR spectrum of Elesesterpene B (**2**) (150 MHz, pyridine‑*d_5_*).

**Figure S11**. DEPT spectrum of Elesesterpene B (**2**).

**Figure S12**. HSQC spectrum of Elesesterpene B (**2**).

**Figure S13**. HMBC spectrum of Elesesterpene B (**2**).

**Figure S14**. ^1^H-^1^H COSY spectrum of Elesesterpene B (**2**).

**Figure S15**. NOESY spectrum of Elesesterpene B (**2**).

**Figure S16**. HR-ESI-MS spectrum of Elesesterpene B (**2**).

**Figure S17**. ^1^H NMR spectrum of Elesesterpene C (**3**) (600 MHz, pyridine‑*d_5_*).

**Figure S18**. ^13^C NMR spectrum of Elesesterpene C (**3**) (150 MHz, pyridine‑*d_5_*).

**Figure S19**. DEPT spectrum of Elesesterpene C (**3**).

**Figure S20**. HSQC spectrum of Elesesterpene C (**3**).

**Figure S21**. HMBC spectrum of Elesesterpene C (**3**).

**Figure S22**. ^1^H-^1^H COSY spectrum of Elesesterpene C (**3**).

**Figure S23**. NOESY spectrum of Elesesterpene C (**3**).

**Figure S24**. HR-ESI-MS spectrum of Elesesterpene C (**3**).

**Figure S25**. ^1^H NMR spectrum of Elesesterpene D (**4**) (600 MHz, pyridine‑*d_5_*).

**Figure S26**. ^13^C NMR spectrum of Elesesterpene D (**4**) (150 MHz, pyridine‑*d_5_*).

**Figure S27**. DEPT spectrum of Elesesterpene D (**4**).

**Figure S28**. HSQC spectrum of Elesesterpene D (**4**).

**Figure S29**. HMBC spectrum of Elesesterpene D (**4**).

**Figure S30**. ^1^H-^1^H COSY spectrum of Elesesterpene D (**4**).

**Figure S31**. NOESY spectrum of Elesesterpene D (**4**).

**Figure S32**. HR-ESI-MS spectrum of Elesesterpene D (**4**).

**Figure S33**. ^1^H NMR spectrum of Elesesterpene E (**5**) (600 MHz, pyridine‑*d_5_*).

**Figure S34**. ^13^C NMR spectrum of Elesesterpene E (**5**) (150 MHz, pyridine‑*d_5_*).

**Figure S35**. DEPT spectrum of Elesesterpene E (**5**).

**Figure S36**. HSQC spectrum of Elesesterpene E (**5**).

**Figure S37**. HMBC spectrum of Elesesterpene E (**5**).

**Figure S38**. ^1^H-^1^H COSY spectrum of Elesesterpene E (**5**).

**Figure S39**. NOESY spectrum of Elesesterpene E (**5**).

**Figure S40**. HR-ESI-MS spectrum of Elesesterpene E (**5**).

**Figure S41**. ^1^H NMR spectrum of Elesesterpene F (**6**) (600 MHz, pyridine‑*d_5_*).

**Figure S42**. ^13^C NMR spectrum of Elesesterpene F (**6**) (150 MHz, pyridine‑*d_5_*).

**Figure S43**. DEPT spectrum of Elesesterpene F (**6**).

**Figure S44**. HSQC spectrum of Elesesterpene F (**6**).

**Figure S45**. HMBC spectrum of Elesesterpene F (**6**).

**Figure S46**. ^1^H-^1^H COSY spectrum of Elesesterpene F (**6**).

**Figure S47**. NOESY spectrum of Elesesterpene F (**6**).

**Figure S48**. HR-ESI-MS spectrum of Elesesterpene F (**6**).

**Figure S49**. ^1^H NMR spectrum of Elesesterpene G (**7**) (600 MHz, pyridine‑*d_5_*).

**Figure S50**. ^13^C NMR spectrum of Elesesterpene G (**7**) (150 MHz, pyridine‑*d_5_*).

**Figure S51**. DEPT spectrum of Elesesterpene G (**7**).

**Figure S52**. HSQC spectrum of Elesesterpene G (**7**).

**Figure S53**. HMBC spectrum of Elesesterpene G (**7**).

**Figure S54**. ^1^H-^1^H COSY spectrum of Elesesterpene G (**7**).

**Figure S55**. NOESY spectrum of Elesesterpene G (**7**).

**Figure S56**. HR-ESI-MS spectrum of Elesesterpene G (**7**).

**Figure S57**. ^1^H NMR spectrum of Elesesterpene H (**8**) (600 MHz, pyridine‑*d_5_*).

**Figure S58**. ^13^C NMR spectrum of Elesesterpene H (**8**) (150 MHz, pyridine‑*d_5_*).

**Figure S59**. DEPT spectrum of Elesesterpene H (**8**).

**Figure S60**. HSQC spectrum of Elesesterpene H (**8**).

**Figure S61**. HMBC spectrum of Elesesterpene H (**8**).

**Figure S62**. ^1^H-^1^H COSY spectrum of Elesesterpene H (**8**).

**Figure S63**. NOESY spectrum of Elesesterpene H (**8**).

**Figure S64**. HR-ESI-MS spectrum of Elesesterpene H (**8**).

**Figure S65**. ^1^H NMR spectrum of Elesesterpene I (**9**) (600 MHz, pyridine‑*d_5_*).

**Figure S66**. ^13^C NMR spectrum of Elesesterpene I (**9**) (150 MHz, pyridine‑*d_5_*).

**Figure S67**. DEPT spectrum of Elesesterpene I (**9**).

**Figure S68**. HSQC spectrum of Elesesterpene I (**9**).

**Figure S69**. HMBC spectrum of Elesesterpene I (**9**).

**Figure S70**. ^1^H-^1^H COSY spectrum of Elesesterpene I (**9**).

**Figure S71**. NOESY spectrum of Elesesterpene I (**9**).

**Figure S72**. HR-ESI-MS spectrum of Elesesterpene I (**9**).

**Figure S73**. ^1^H NMR spectrum of Elesesterpene J (**10**) (600 MHz, pyridine‑*d_5_*).

**Figure S74**. ^13^C NMR spectrum of Elesesterpene J (**10**) (150 MHz, pyridine‑*d_5_*).

**Figure S75**. DEPT spectrum of Elesesterpene J (**10**).

**Figure S76**. HSQC spectrum of Elesesterpene J (**10**).

**Figure S77**. HMBC spectrum of Elesesterpene J (1**0**).

**Figure S78**. ^1^H-^1^H COSY spectrum of Elesesterpene J (**10**).

**Figure S79**. NOESY spectrum of Elesesterpene J (**10**).

**Figure S80**. HR-ESI-MS spectrum of Elesesterpene J (**10**).

**Figure S81**. ^1^H NMR spectrum of Elesesterpene K (**11**) (600 MHz, pyridine‑*d_5_*).

**Figure S82**. ^13^C NMR spectrum of Elesesterpene K (**11**) (150 MHz, pyridine‑*d_5_*).

**Figure S83**. DEPT spectrum of Elesesterpene K (**11**).

**Figure S84**. HSQC spectrum of Elesesterpene K (**11**).

**Figure S85**. HMBC spectrum of Elesesterpene K (**11**).

**Figure S86**. ^1^H-^1^H COSY spectrum of Elesesterpene K (**11**).

**Figure S87**. NOESY spectrum of Elesesterpene K (**11**).

**Figure S88**. HR-ESI-MS spectrum of Elesesterpene K (**11**).

**Figure S89**. Gas chromatogram of the L-Rhamnose, D-Glucose, compounds **3**, **4**, **5**, and **11**.

**Supplementary Data**

***Elesesterpene A (1)*:** white acicular crystal; mp 354-355 ℃; ${[]}_{D}^{24}$ = + 272.0 (c = 0.02, MeOH); UV (MeOH) *λ*_max_ 200.4 nm; HR-ESI-MS: *m/z* 503.3360 [M + H] ^+^, (calcd for C_30_H_47_O_6_, 503.3373). The ^1^H (pyridine‑*d_5_*, 600 MHz) and ^13^C NMR (pyridine‑*d_5_*, 150 MHz) data, see **Table 1**.

***Elesesterpene B (2)*:** white acicular crystal; mp 338-339 ℃; ${[]}_{D}^{24}$ = - 45.0 (c = 0.04, MeOH); UV (MeOH) *λ*_max_ 199.2 nm; HR-ESI-MS: *m/z* 485.3260 [M + H] ^+^, (calcd for C_30_H_45_O_5_, 485.3267). The ^1^H (pyridine‑*d_5_*, 600 MHz) and ^13^C NMR (pyridine‑*d_5_*, 150 MHz) data, see **Table 1**.

***Elesesterpene C (3)*:** yellow amorphous solid; ${[]}_{D}^{24}$ = - 20 (c = 0.1, MeOH); UV (MeOH) *λ*_max_ 199.7 nm; HR-ESI-MS: *m/z* 985.5359 [M + H] ^+^, (calcd for C_50_H_81_O_19_, 985.5372). The ^1^H (pyridine‑*d_5_*, 600 MHz) and ^13^C NMR (pyridine‑*d_5_*, 150 MHz) data, see **Table 2**.

***Elesesterpene D (4)*:** yellow amorphous solid; ${[]}_{D}^{24}$ = - 24 (c = 0.1, MeOH); UV (MeOH) *λ*_max_ 203.2 nm; HR-ESI-MS: *m/z* 972.5525 [M + NH_4_] ^+^, (calcd for C_49_H_82_NO_18_, 972.5532). The ^1^H (pyridine‑*d_5_*, 600 MHz) and ^13^C NMR (pyridine‑*d_5_*, 150 MHz) data, see **Table 2**.

***Elesesterpene E (5)*:** yellow amorphous solid; ${[]}_{D}^{24}$ = - 34 (c = 0.1, MeOH); UV (MeOH) *λ*_max_ 204.4 nm; HR-ESI-MS: *m/z* 986.5679 [M + NH_4_] ^+^, (calcd for C_50_H_84_NO_18_, 986.5688). The ^1^H (pyridine‑*d_5_*, 600 MHz) and ^13^C NMR (pyridine‑*d_5_*, 150 MHz) data, see **Table 2**.

***Elesesterpene F (6)*:** colorless amorphous solid; ${[]}_{D}^{24}$ = - 40 (c = 0.13, MeOH); UV (MeOH) *λ*_max_ 198.0 nm; HR-ESI-MS: *m/z* 517.3179 [M + H] ^+^, (calcd for C_30_H_45_O_7_, 517.3165). The ^1^H (pyridine‑*d_5_*, 600 MHz) and ^13^C NMR (pyridine‑*d_5_*, 150 MHz) data, see **Table 1**.

***Elesesterpene G (7)*:** Yellow amorphous solid; ${[]}_{D}^{24}$ = + 57.8 (c = 0.14, MeOH); UV (MeOH) *λ*_max_ 199.2 nm; HR-ESI-MS: *m/z* 487.3074 [M + H] ^+^, (calcd for C_29_H_43_O_6_, 487.3060). The ^1^H (pyridine‑*d_5_*, 600 MHz) and ^13^C NMR (pyridine‑*d_5_*, 150 MHz) data, see **Table 1**.

***Elesesterpene H (8)*:** yellow amorphous solid; ${[]}_{D}^{24}$ = + 55.0 (c = 0.16, MeOH); UV (MeOH) *λ*_max_ 195.7 nm; HR-ESI-MS: *m/z* 533.3467 [M + H] ^+^, (calcd for C_31_H_49_O_7_, 533.3478). The ^1^H (pyridine‑*d_5_*, 600 MHz) and ^13^C NMR (pyridine‑*d_5_*, 150 MHz) data, see **Table 3**.

***Elesesterpene I (9)*:** yellow amorphous solid; ${[]}_{D}^{24}$ = + 85.5 (c = 0.14, MeOH); UV (MeOH) *λ*_max_ 201.5 nm; HR-ESI-MS: *m/z* 547.3627 [M + H] ^+^, (calcd for C_32_H_51_O_7_, 547.3635). The ^1^H (pyridine‑*d_5_*, 600 MHz) and ^13^C NMR (pyridine‑*d_5_*, 150 MHz) data, see **Table 3**.

***Elesesterpene J (10)*:** white amorphous powder; ${[]}_{D}^{24}$ = + 78.4 (c = 0.12, MeOH); UV (MeOH) *λ*_max_ 200.4 nm; HR-ESI-MS: *m/z* 503.3366 [M + H] ^+^, (calcd for C_30_H_47_O_6_, 503.3373). The ^1^H (pyridine‑*d_5_*, 600 MHz) and ^13^C NMR (pyridine‑*d_5_*, 150 MHz) data, see **Table 3**.

***Elesesterpene K (11)*:** yellow amorphous solid; ${[]}_{D}^{24}$ = - 22 (c = 0.1, MeOH); UV (MeOH) *λ*_max_ 198.0 nm; HR-ESI-MS: *m/z* 1001.5293 [M + H] ^+^, (calcd for C_50_H_81_O_20_, 1001.5321). The ^1^H (pyridine‑*d_5_*, 600 MHz) and ^13^C NMR (pyridine‑*d_5_*, 150 MHz) data, see **Table 3**.

**Supplementary Figures**

**Figure S1**. ^1^H NMR spectrum of Elesesterpene A (**1**) (600 MHz, pyridine‑*d_5_*).
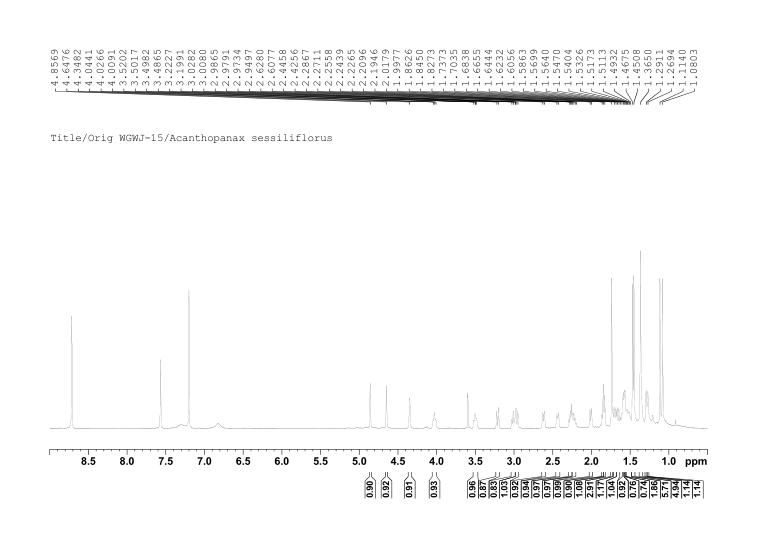


**Figure S2**. ^13^C NMR spectrum of Elesesterpene A (**1**) (150 MHz, pyridine‑*d_5_*).
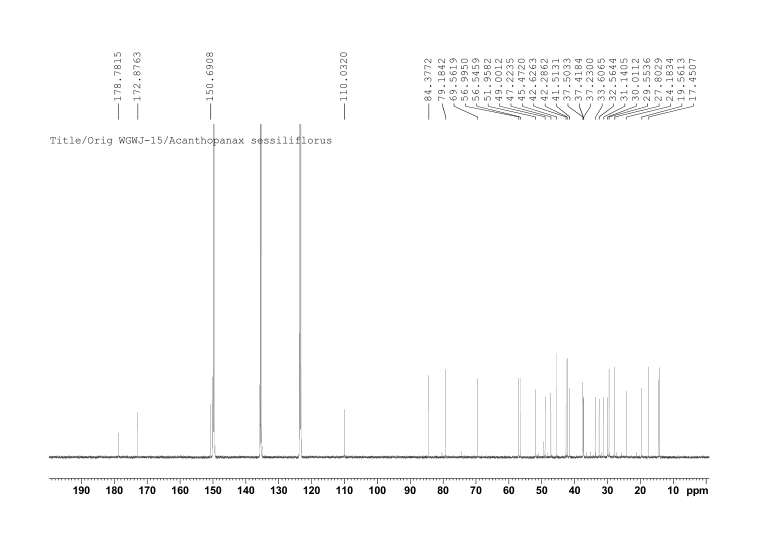


**Figure S3**. DEPT spectrum of Elesesterpene A (**1**).
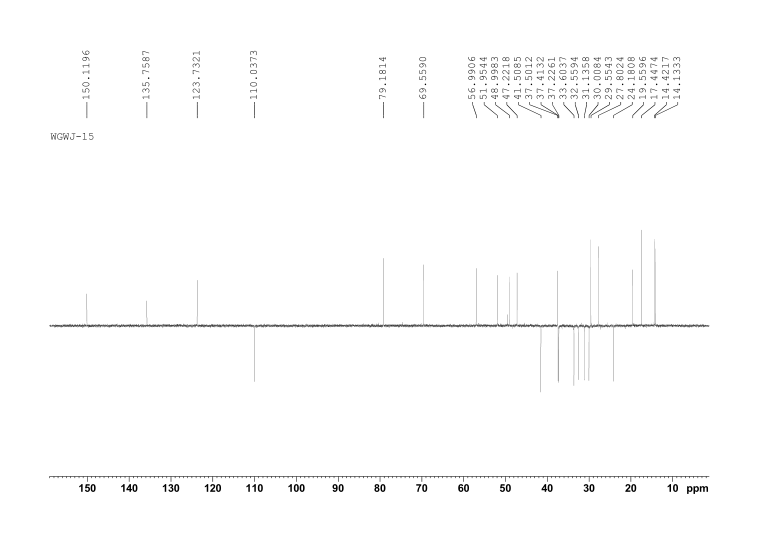


**Figure S4**. HSQC spectrum of Elesesterpene A (**1**).
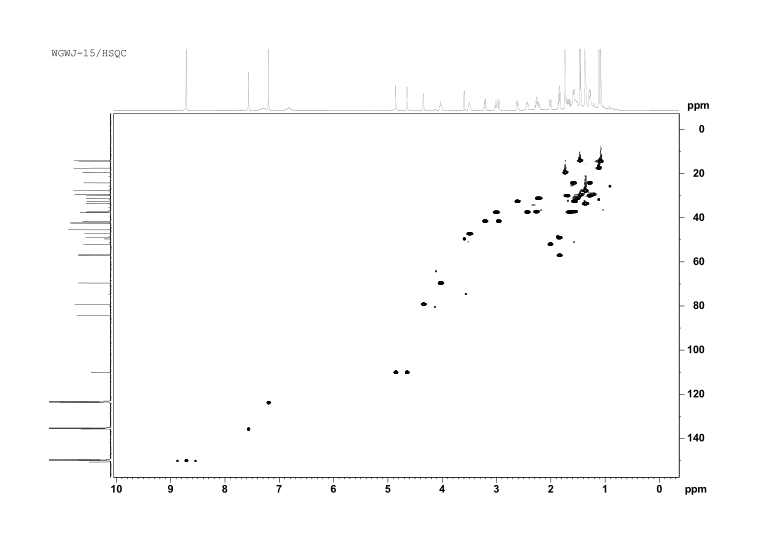


**Figure S5**. HMBC spectrum of Elesesterpene A (**1**).
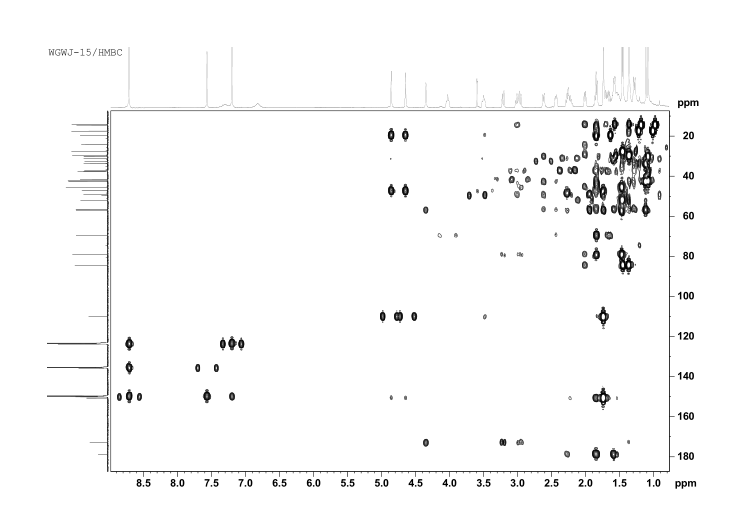


**Figure S6**. ^1^H-^1^H COSY spectrum of Elesesterpene A (**1**).
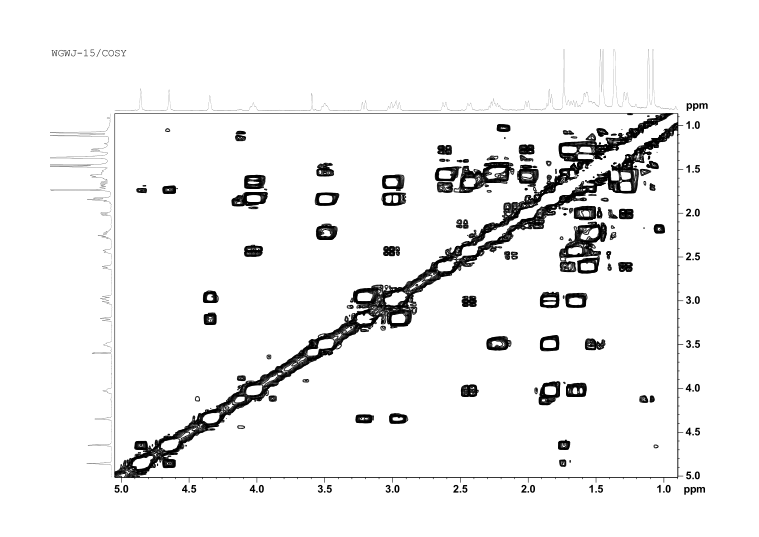


**Figure S7**. NOESY spectrum of Elesesterpene A (**1**).
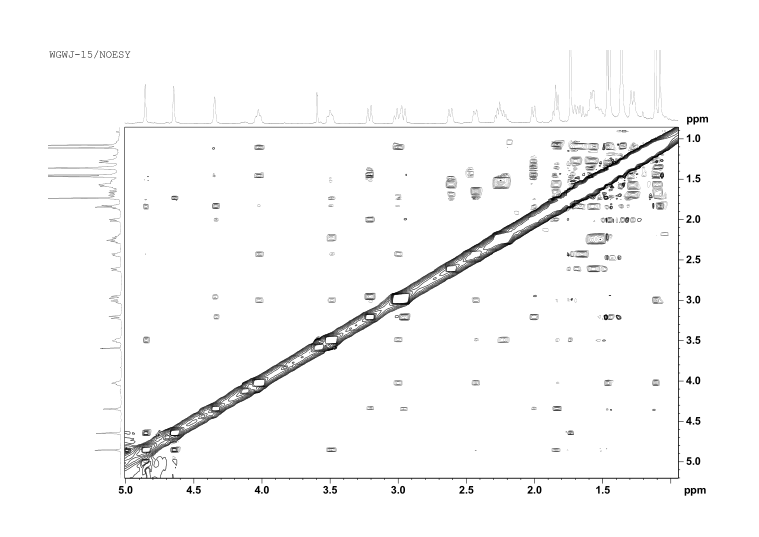


**Figure S8**. HR-ESI-MS spectrum of Elesesterpene A (**1**).

**Figure S9**. ^1^H NMR spectrum of Elesesterpene B (**2**) (600 MHz, pyridine‑*d_5_*).
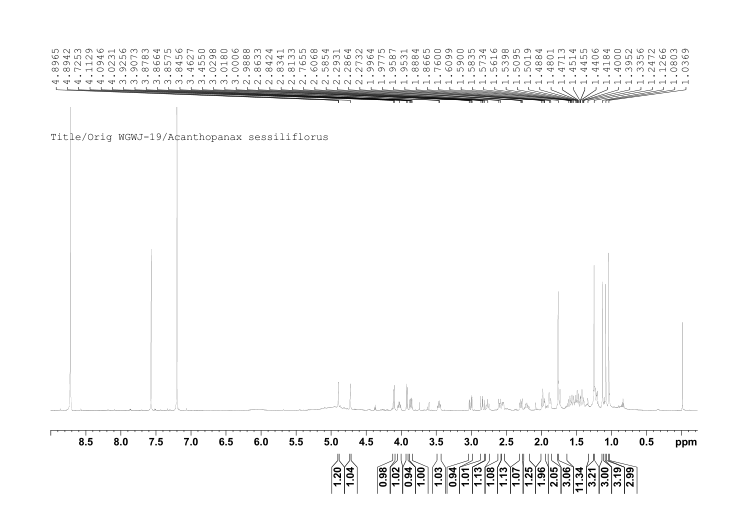


**Figure S10**. ^13^C NMR spectrum of Elesesterpene B (**2**) (150 MHz, pyridine‑*d_5_*).
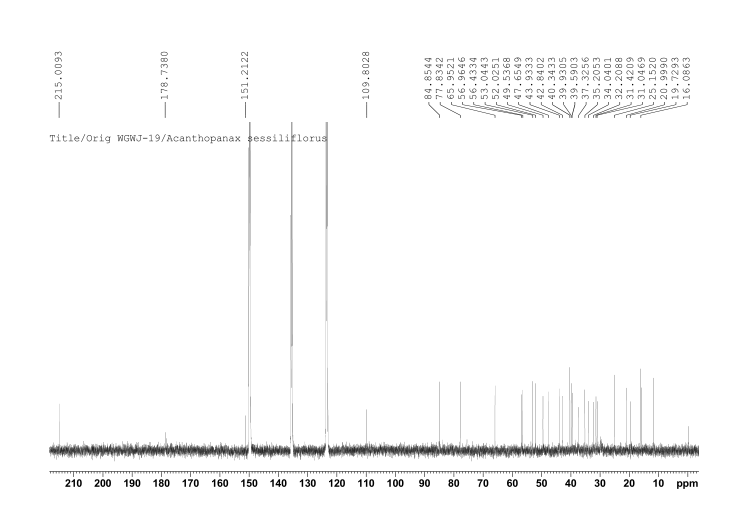


**Figure S11**. DEPT spectrum of Elesesterpene B (**2**).
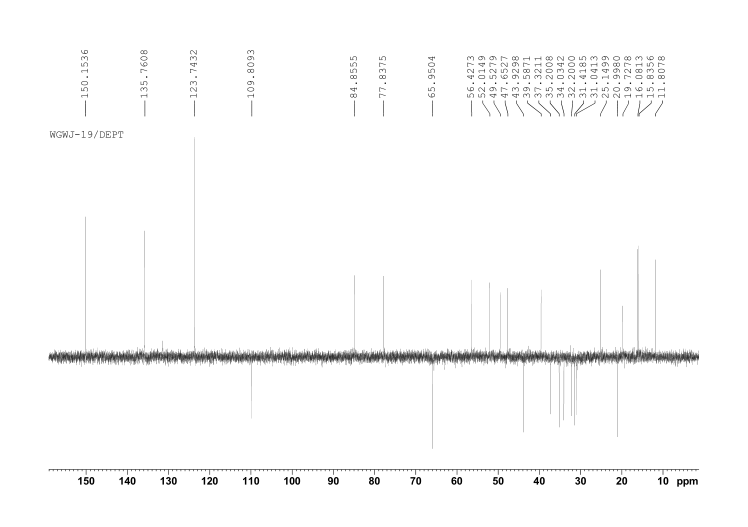


**Figure S12**. HSQC spectrum of Elesesterpene B (**2**).
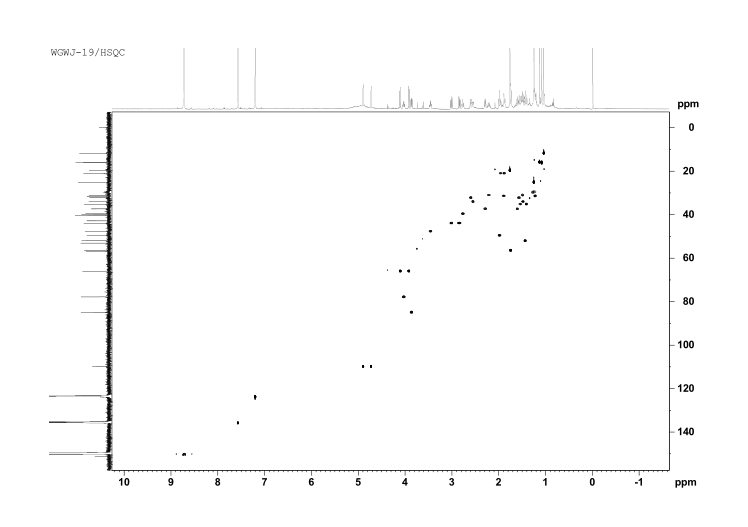


**Figure S13**. HMBC spectrum of Elesesterpene B (**2**).
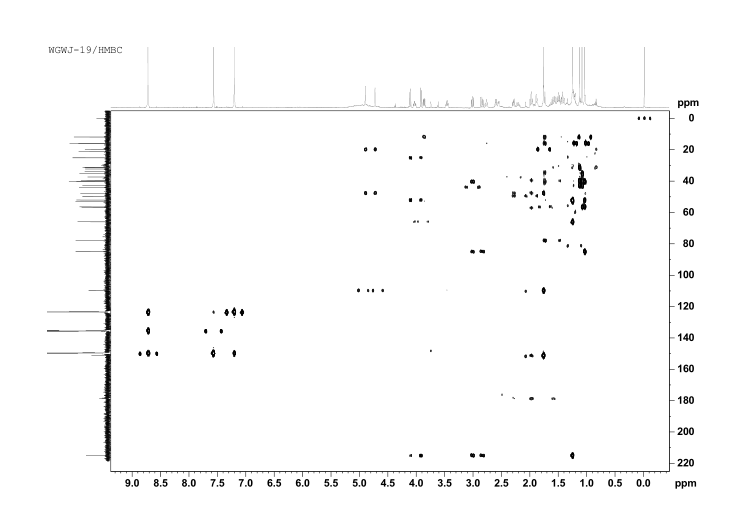


**Figure S14**. ^1^H-^1^H COSY spectrum of Elesesterpene B (**2**).
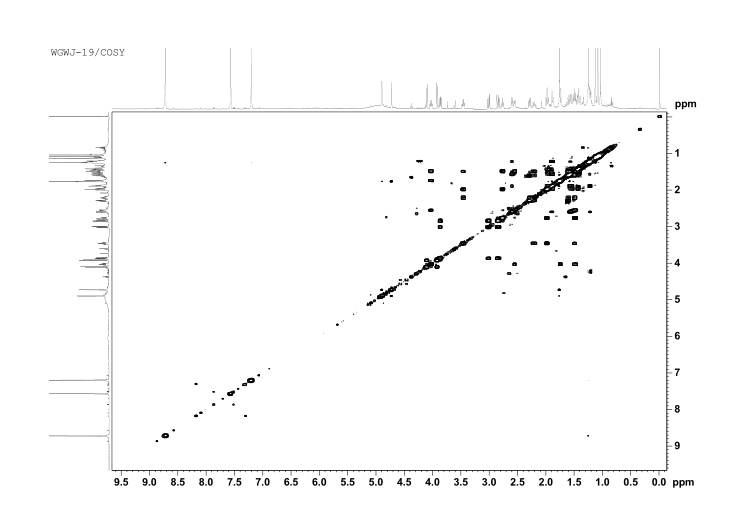


**Figure S15**. NOESY spectrum of Elesesterpene B (**2**).
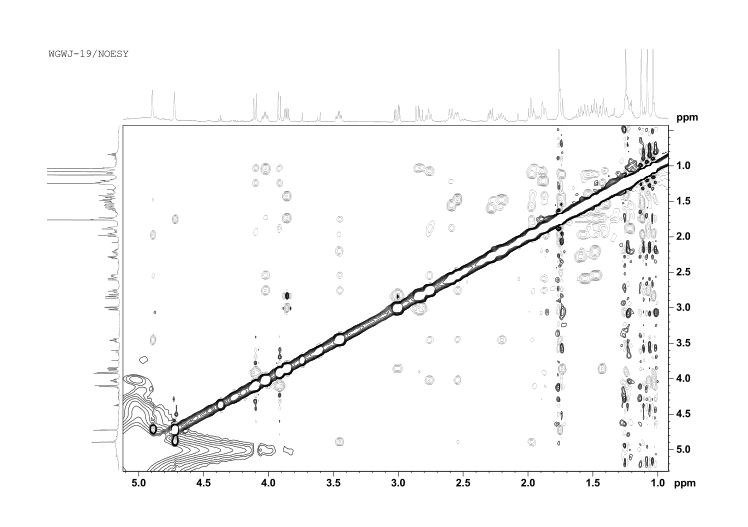


**Figure S16**. HR-ESI-MS spectrum of Elesesterpene B (**2**).

**Figure S17**. ^1^H NMR spectrum of Elesesterpene C (**3**) (600 MHz, pyridine‑*d_5_*).
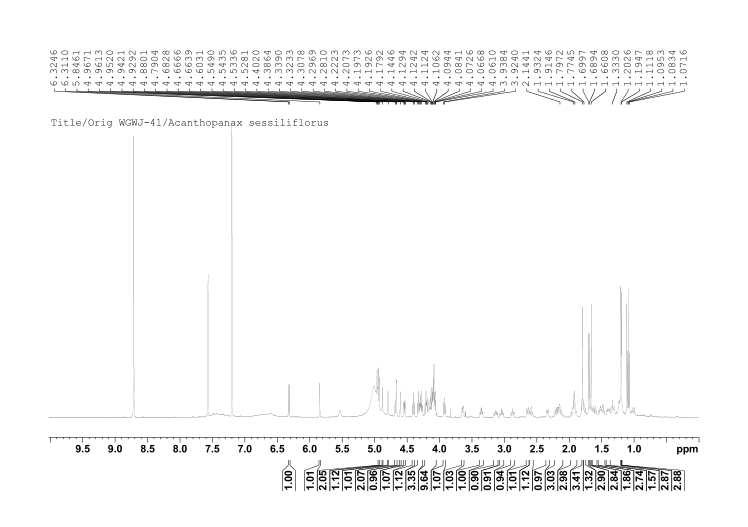


**Figure S18**. ^13^C NMR spectrum of Elesesterpene C (**3**) (150 MHz, pyridine‑*d_5_*).
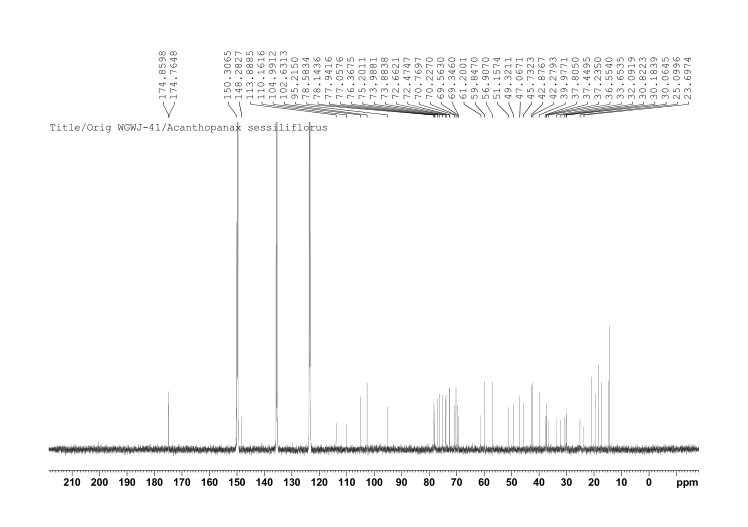


**Figure S19**. DEPT spectrum of Elesesterpene C (**3**).
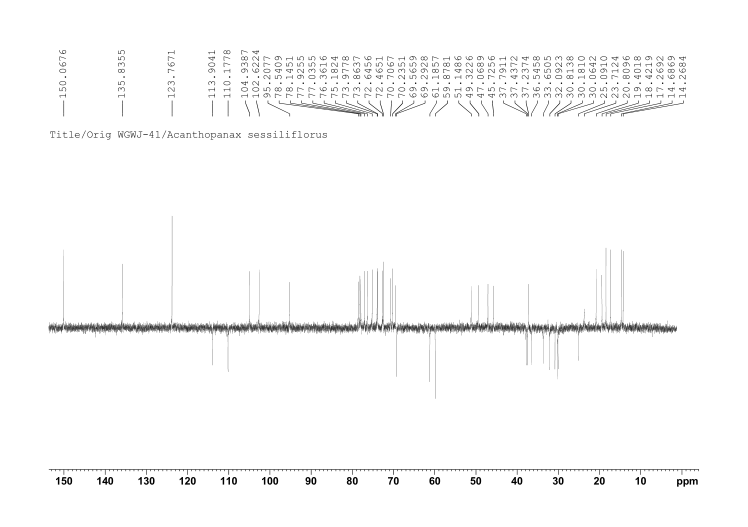


**Figure S20**. HSQC spectrum of Elesesterpene C (**3**).
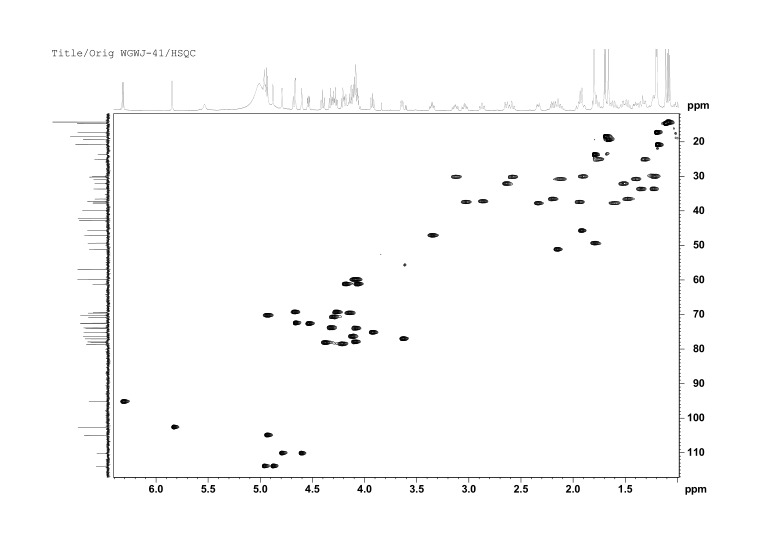


**Figure S21**. HMBC spectrum of Elesesterpene C (**3**).
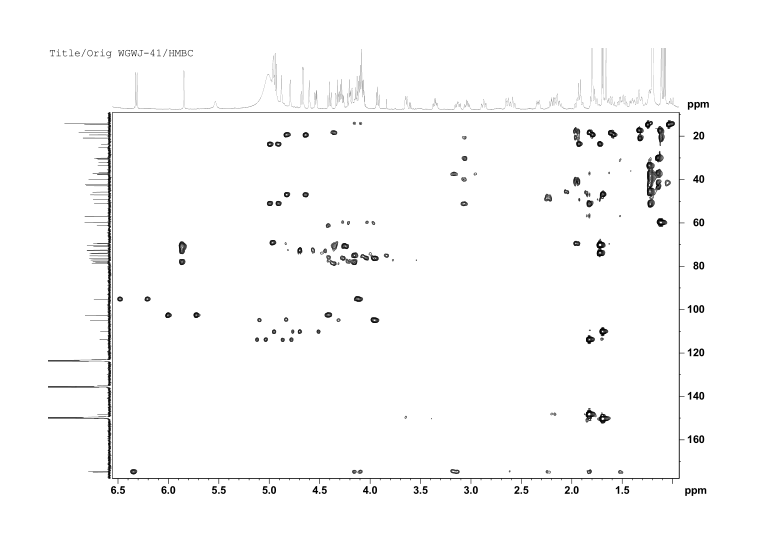


**Figure S22**. ^1^H-^1^H COSY spectrum of Elesesterpene C (**3**).
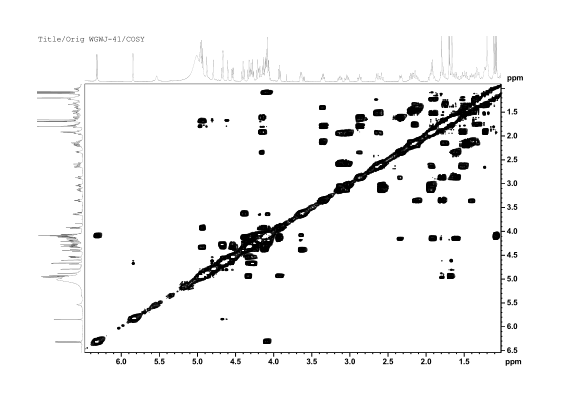


**Figure S23**. NOESY spectrum of Elesesterpene C (**3**).
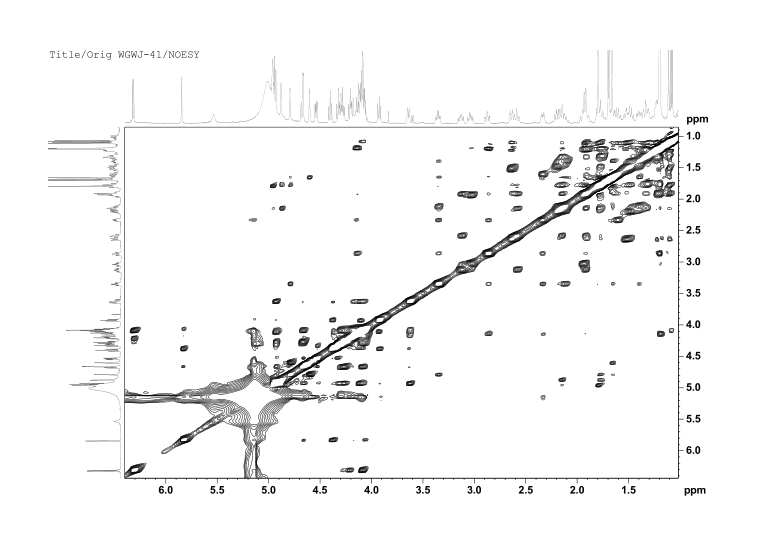


**Figure S24**. HR-ESI-MS spectrum of Elesesterpene C (**3**).

**Figure S25**. ^1^H NMR spectrum of Elesesterpene D (**4**) (600 MHz, pyridine‑*d_5_*).
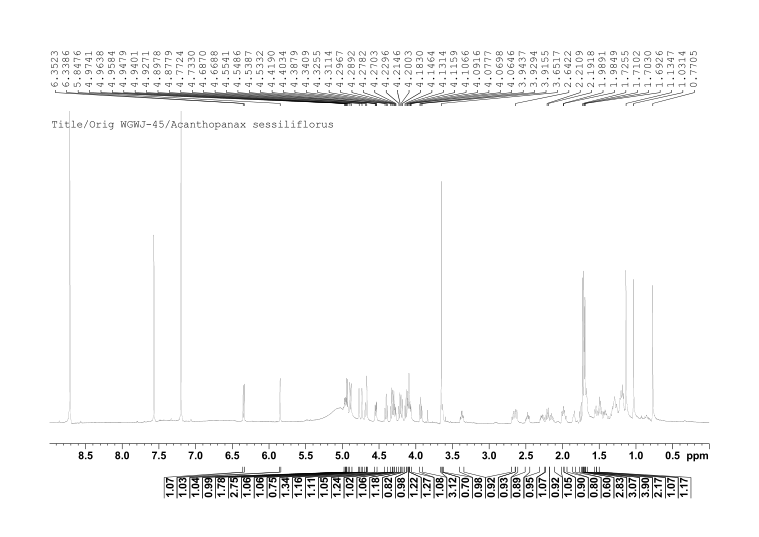


**Figure S26**. ^13^C NMR spectrum of Elesesterpene D (**4**) (150 MHz, pyridine‑*d_5_*).
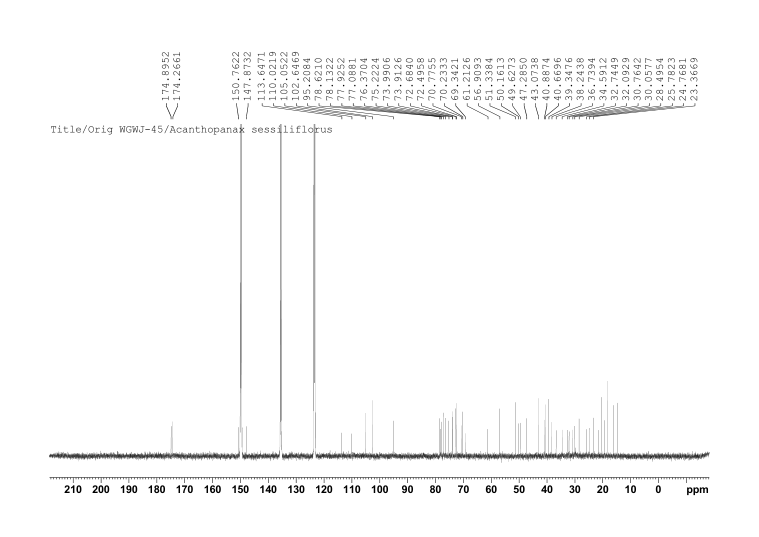


**Figure S27**. DEPT spectrum of Elesesterpene D (**4**).
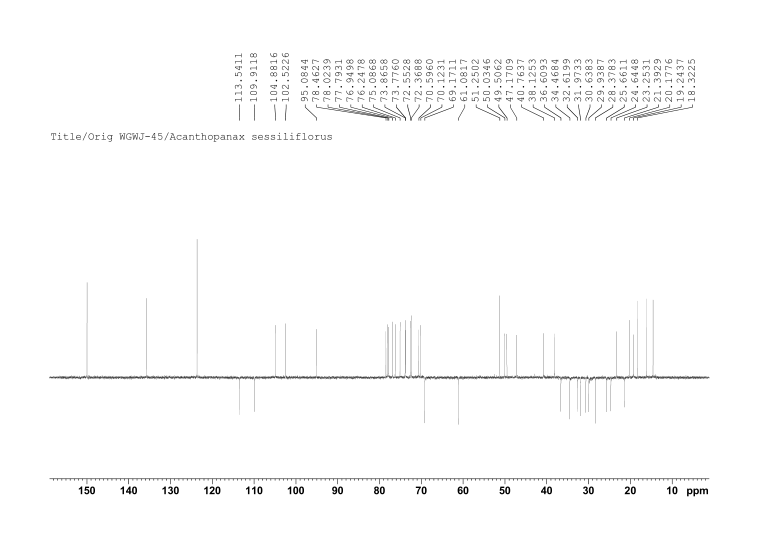


**Figure S28**. HSQC spectrum of Elesesterpene D (**4**).
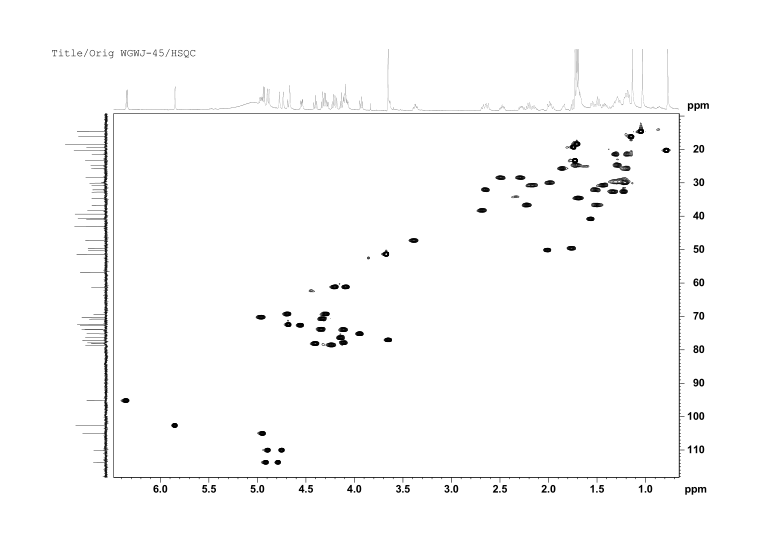


**Figure S29**. HMBC spectrum of Elesesterpene D (**4**).
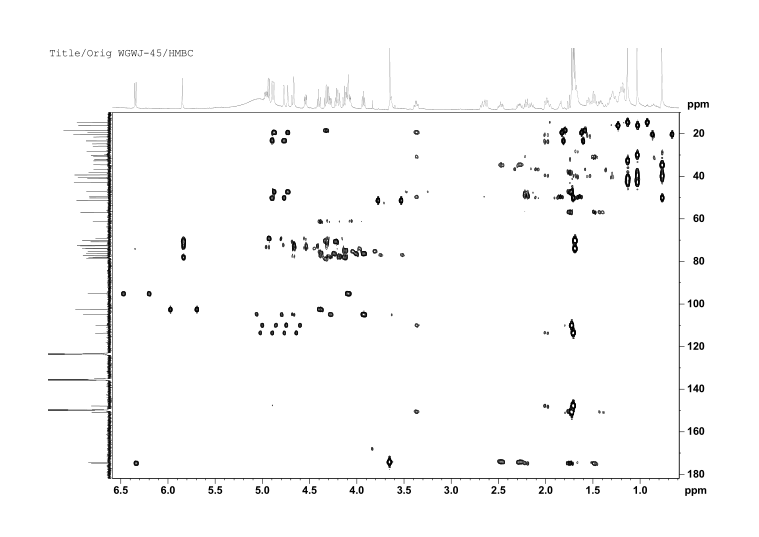


**Figure S30**. ^1^H-^1^H COSY spectrum of Elesesterpene D (**4**).
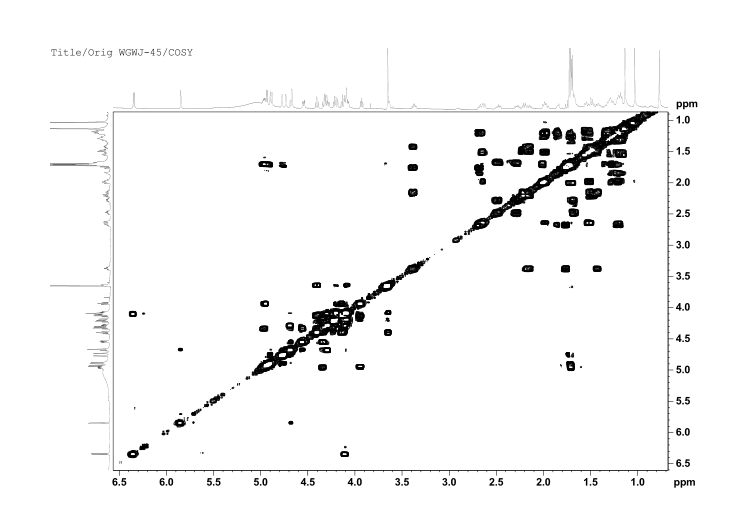


**Figure S31**. NOESY spectrum of Elesesterpene D (**4**).
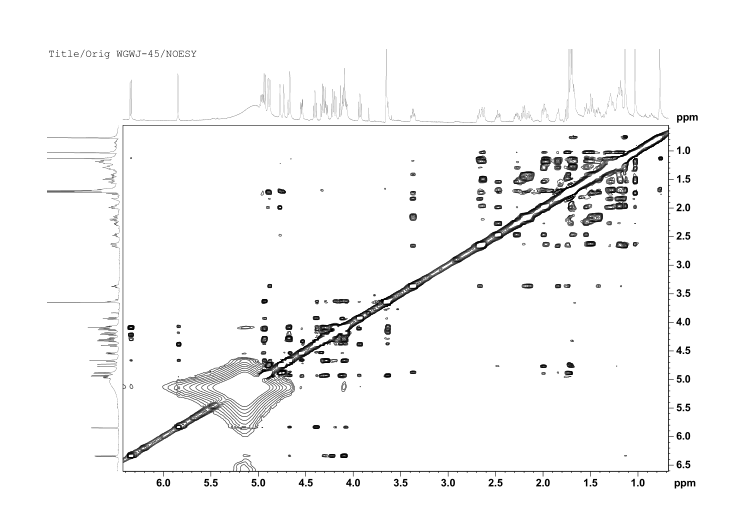


**Figure S32**. HR-ESI-MS spectrum of Elesesterpene D (**4**).

**Figure S33**. ^1^H NMR spectrum of Elesesterpene E (**5**) (600 MHz, pyridine‑*d_5_*).
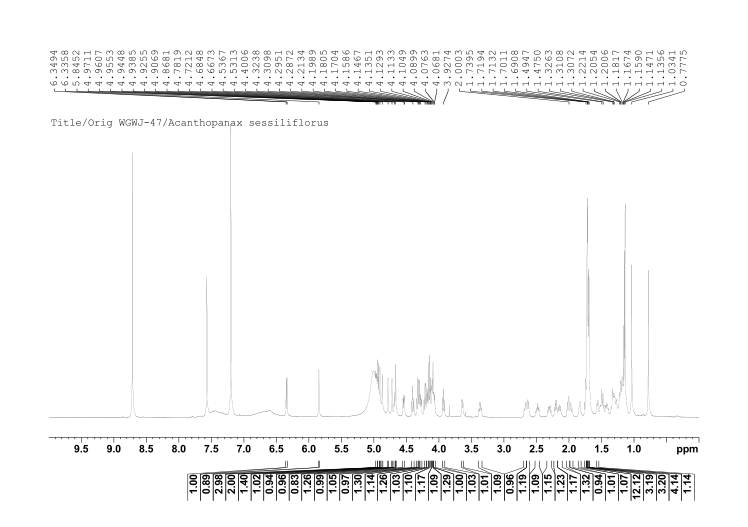


**Figure S34**. ^13^C NMR spectrum of Elesesterpene E (**5**) (150 MHz, pyridine‑*d_5_*).
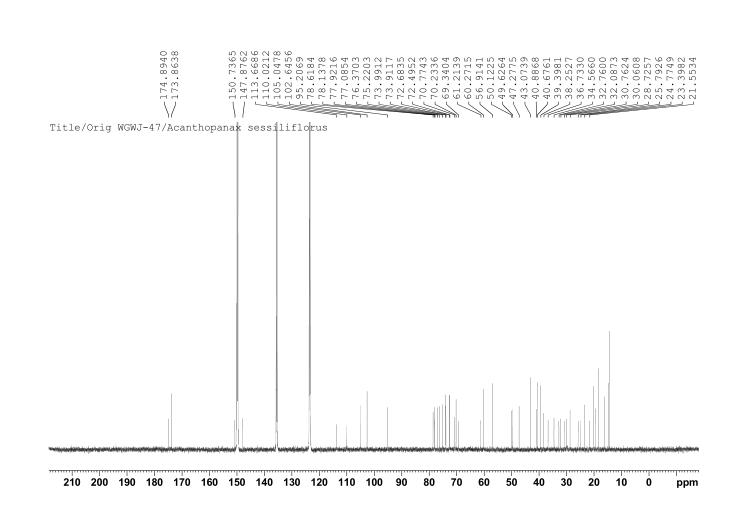


**Figure S35**. DEPT spectrum of Elesesterpene E (**5**).
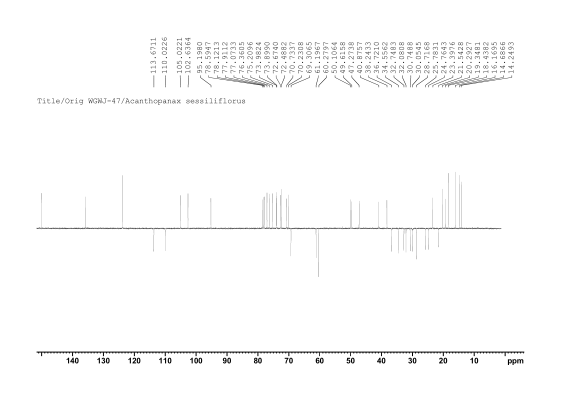


**Figure S36**. HSQC spectrum of Elesesterpene E (**5**).
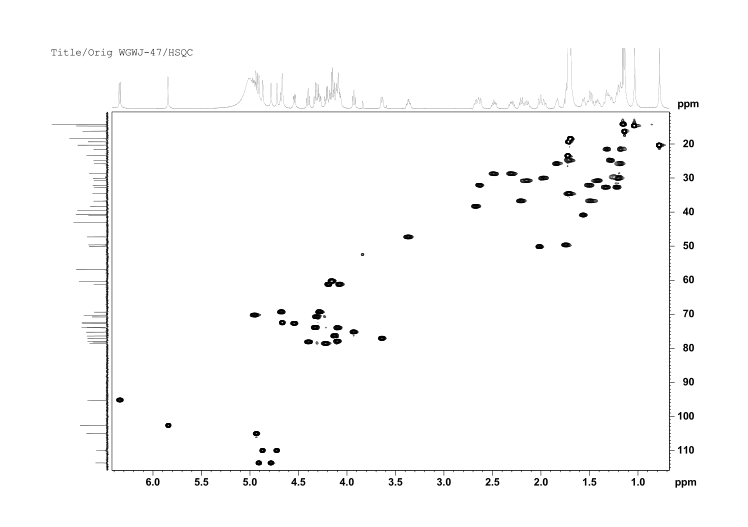


**Figure S37**. HMBC spectrum of Elesesterpene E (**5**).
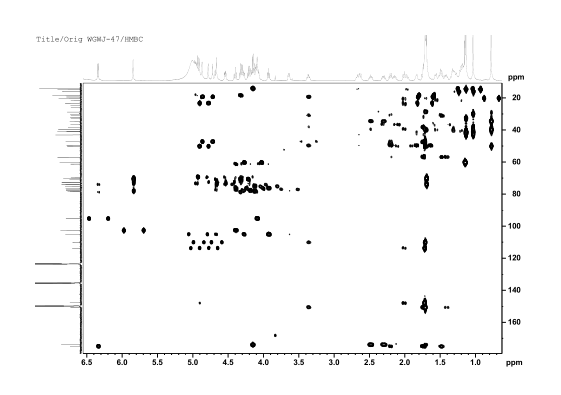


**Figure S38**. ^1^H-^1^H COSY spectrum of Elesesterpene E (**5**).
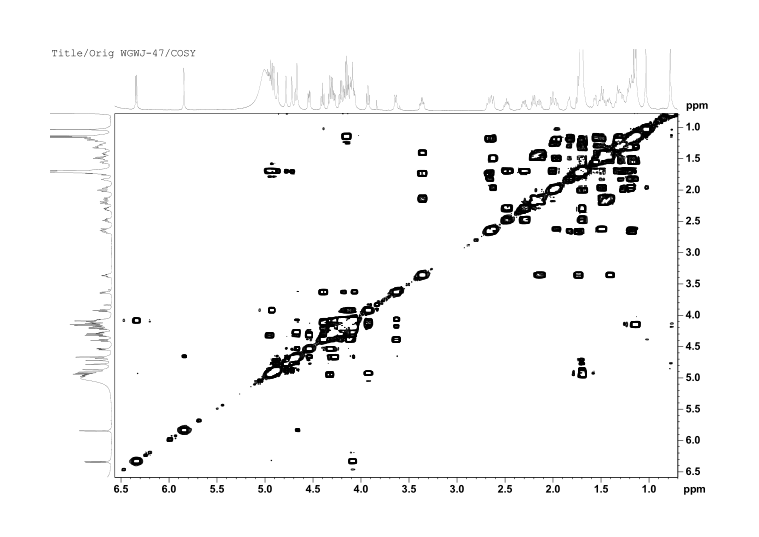


**Figure S39**. NOESY spectrum of Elesesterpene E (**5**).
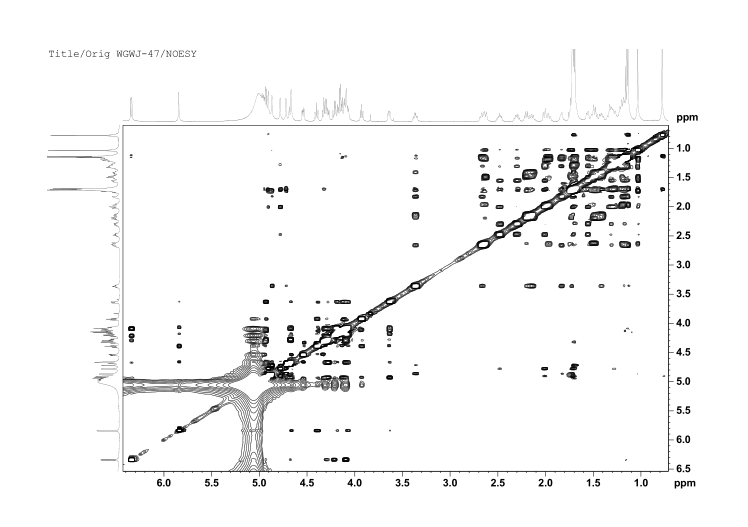


**Figure S40**. HR-ESI-MS spectrum of Elesesterpene E (**5**).

**Figure S41**. ^1^H NMR spectrum of Elesesterpene F (**6**) (600 MHz, pyridine‑*d_5_*).
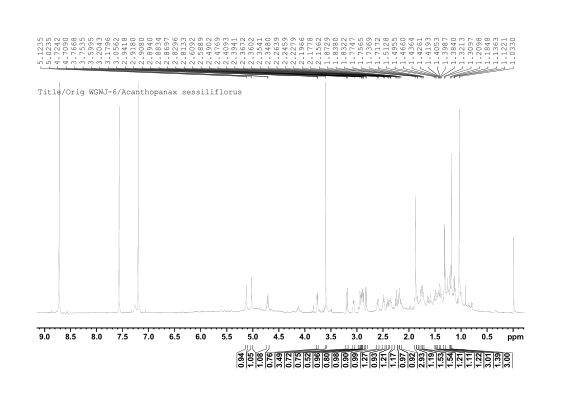


**Figure S42**. ^13^C NMR spectrum of Elesesterpene F (**6**) (150 MHz, pyridine‑*d_5_*).
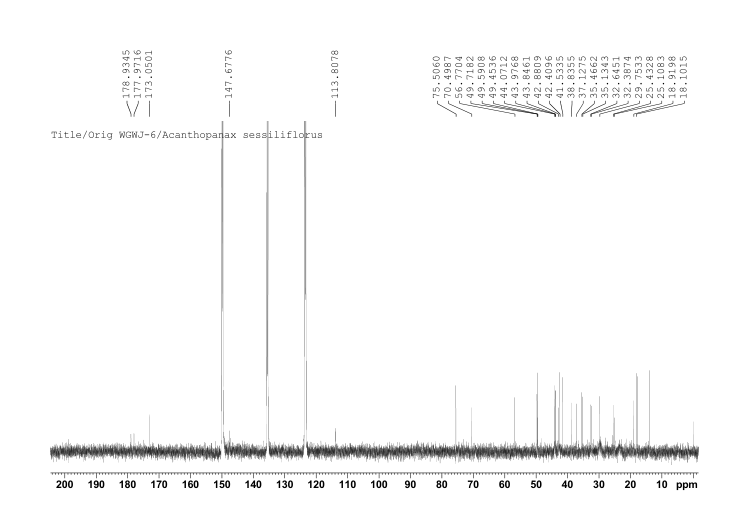


**Figure S43**. DEPT spectrum of Elesesterpene F (**6**).
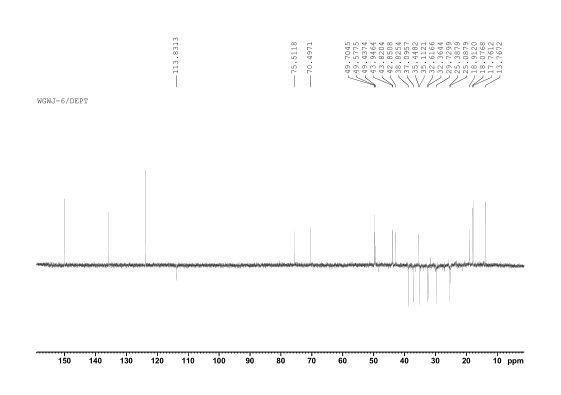


**Figure S44**. HSQC spectrum of Elesesterpene F (**6**).
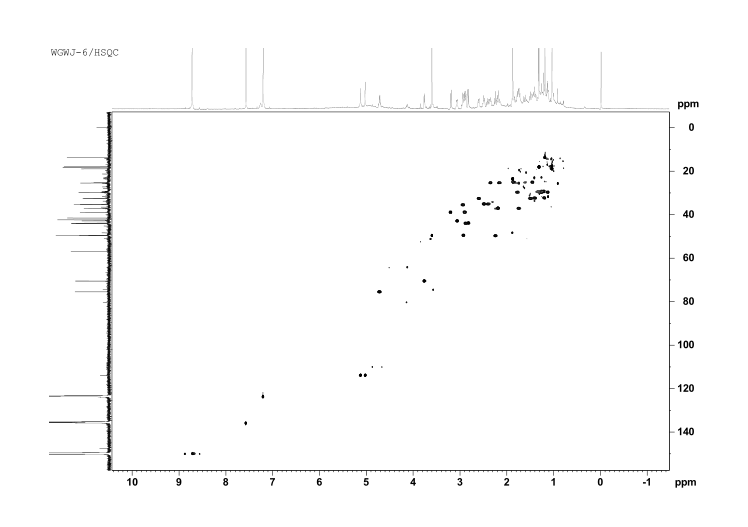


**Figure S45**. HMBC spectrum of Elesesterpene F (**6**).
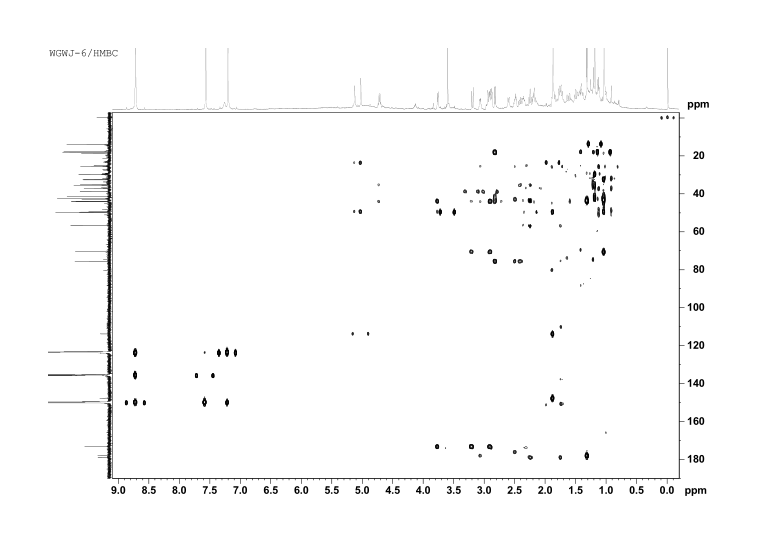


**Figure S46**. ^1^H-^1^H COSY spectrum of Elesesterpene F (**6**).
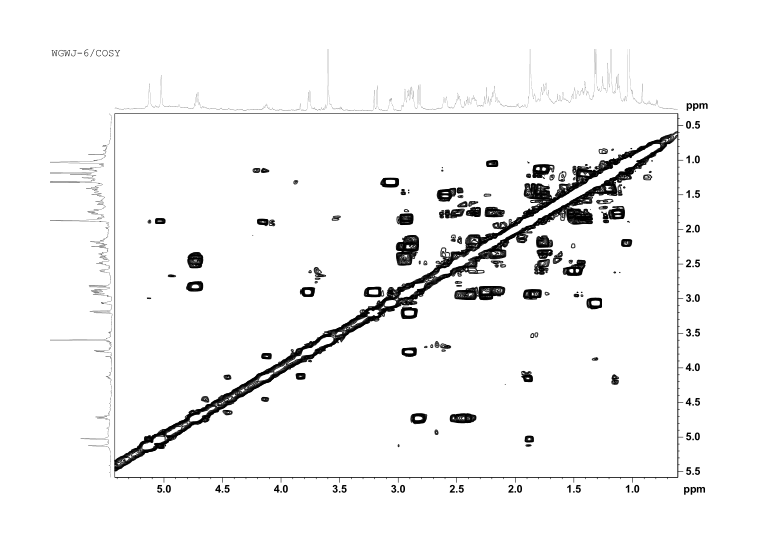


**Figure S47**. NOESY spectrum of Elesesterpene F (**6**).
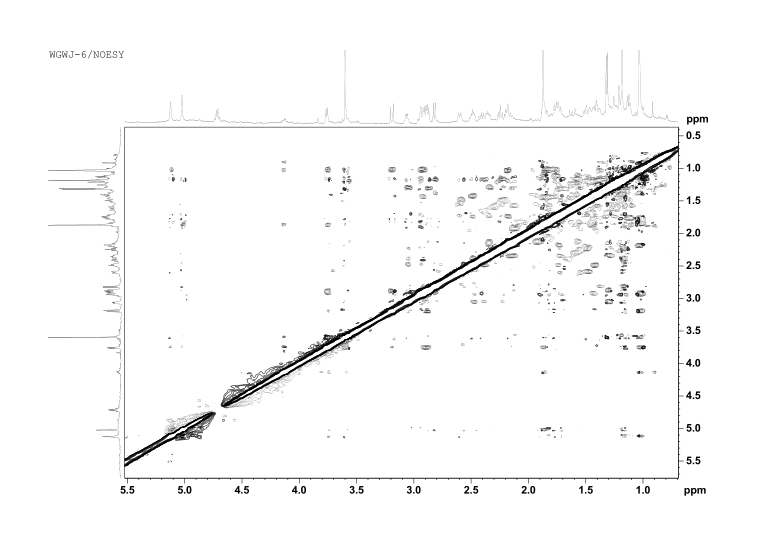


**Figure S48**. HR-ESI-MS spectrum of Elesesterpene F (**6**).

**Figure S49**. ^1^H NMR spectrum of Elesesterpene G (**7**) (600 MHz, pyridine‑*d_5_*).
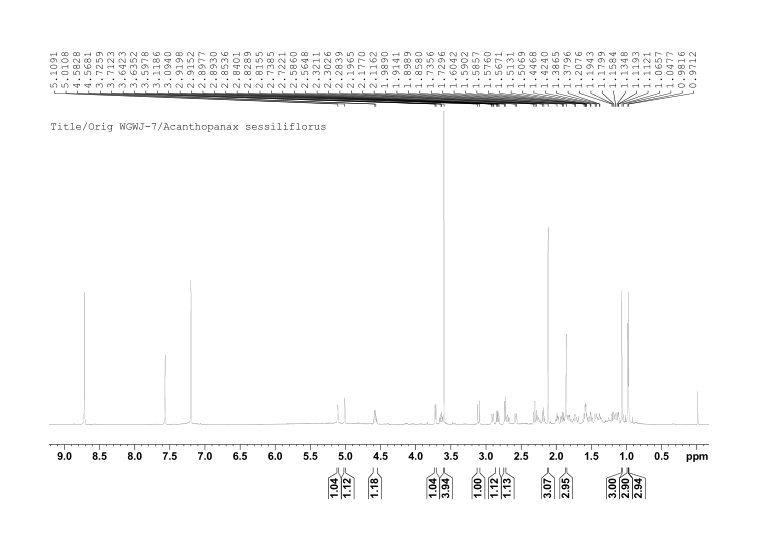


**Figure S50**. ^13^C NMR spectrum of Elesesterpene G (**7**) (150 MHz, pyridine‑*d_5_*).
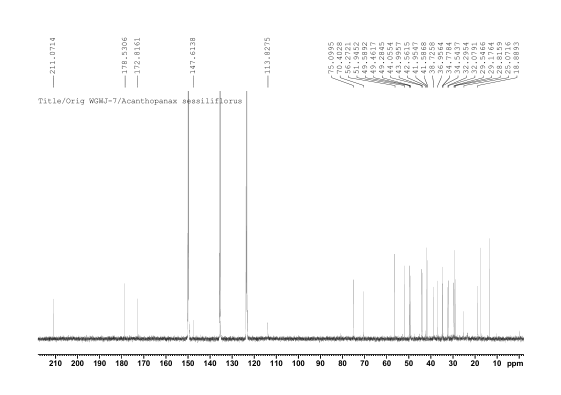


**Figure S51**. DEPT spectrum of Elesesterpene G (**7**).
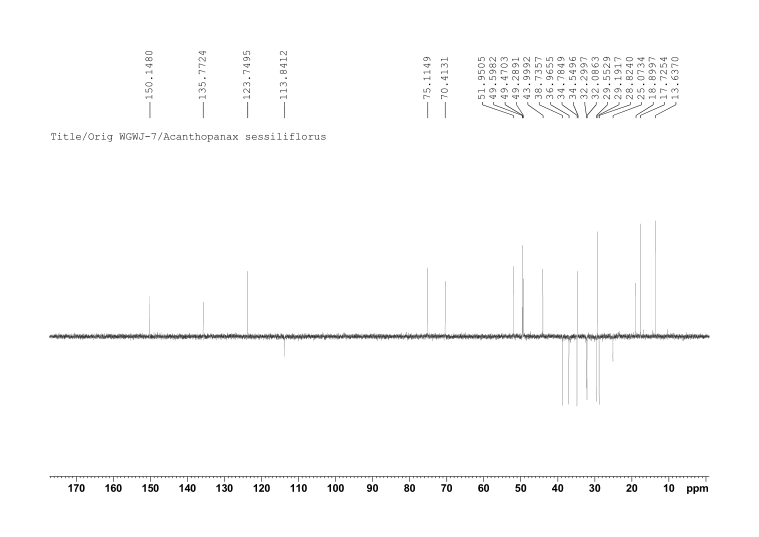


**Figure S52**. HSQC spectrum of Elesesterpene G (**7**).
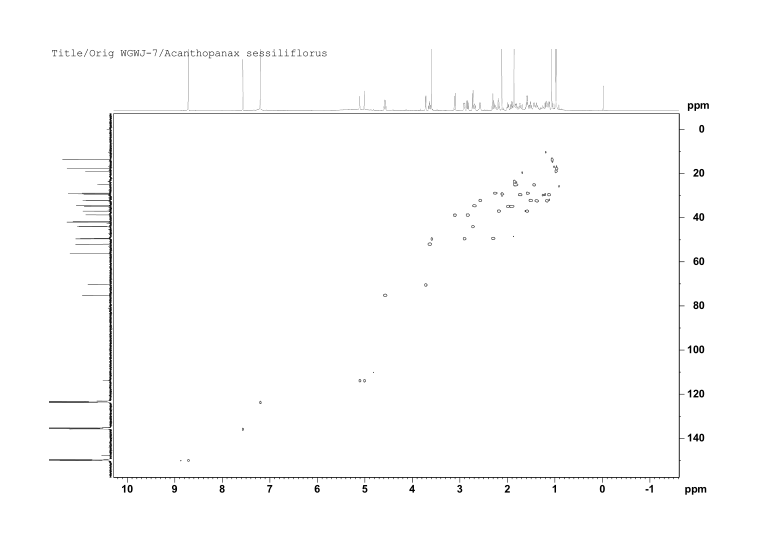


**Figure S53**. HMBC spectrum of Elesesterpene G (**7**).
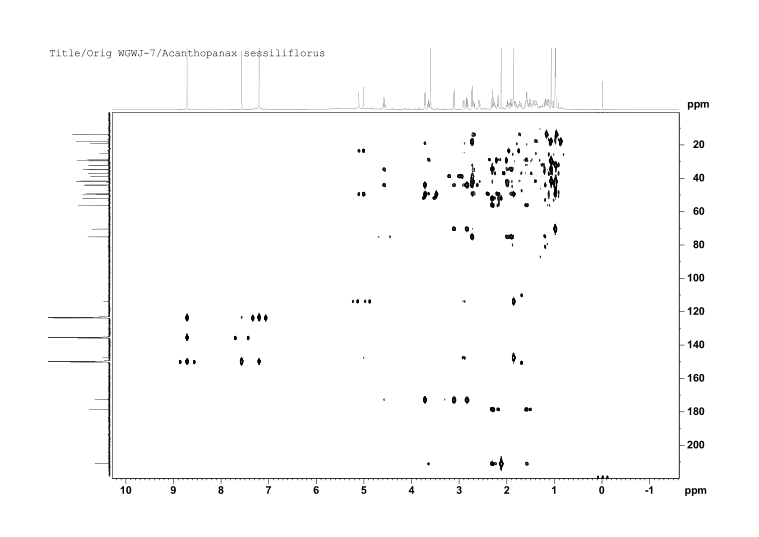


**Figure S54**. ^1^H-^1^H COSY spectrum of Elesesterpene G (**7**).
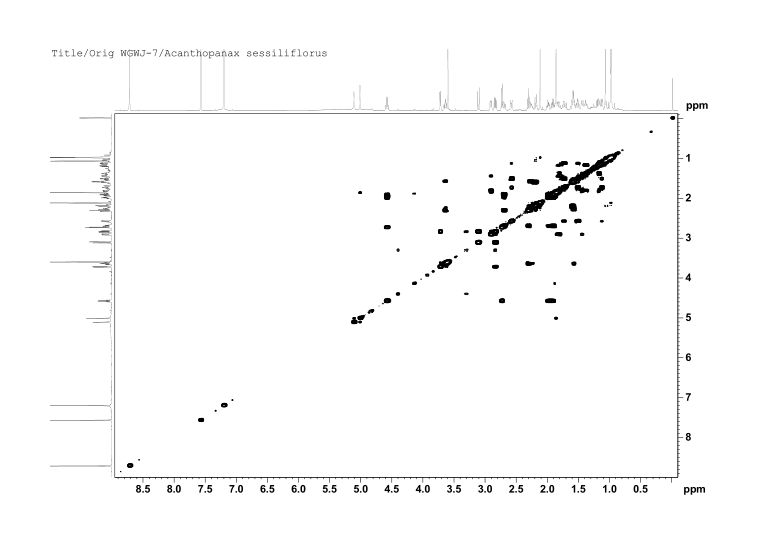


**Figure S55**. NOESY spectrum of Elesesterpene G (**7**).
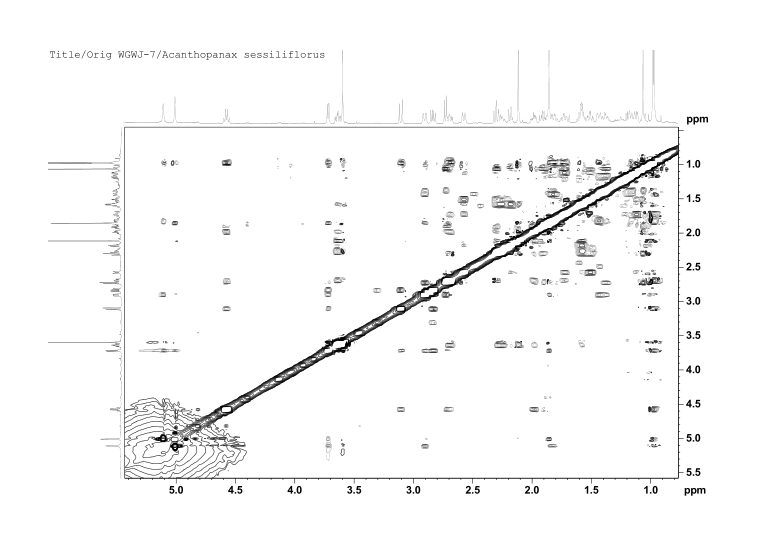


**Figure S56**. HR-ESI-MS spectrum of Elesesterpene G (**7**).

**Figure S57**. ^1^H NMR spectrum of Elesesterpene H (**8**) (600 MHz, pyridine‑*d_5_*).
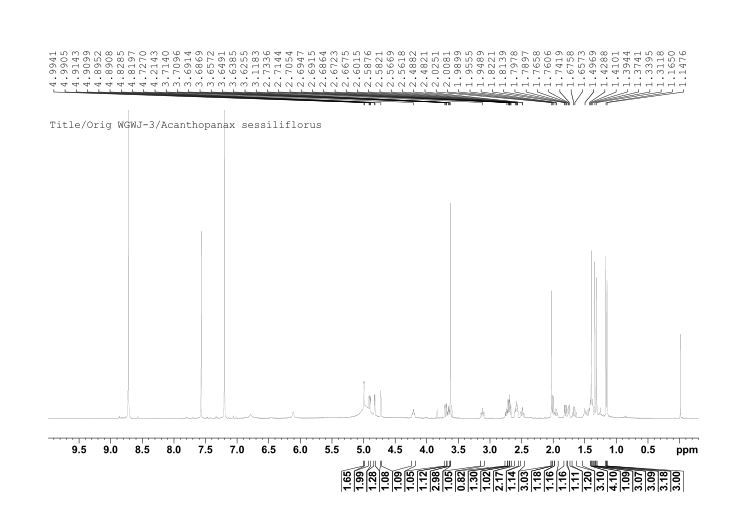


**Figure S58**. ^13^C NMR spectrum of Elesesterpene H (**8**) (150 MHz, pyridine‑*d_5_*).
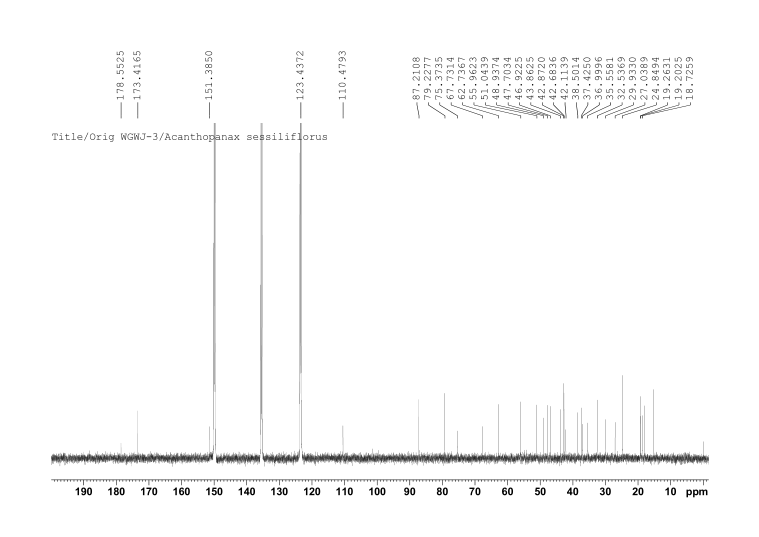


**Figure S59**. DEPT spectrum of Elesesterpene H (**8**).
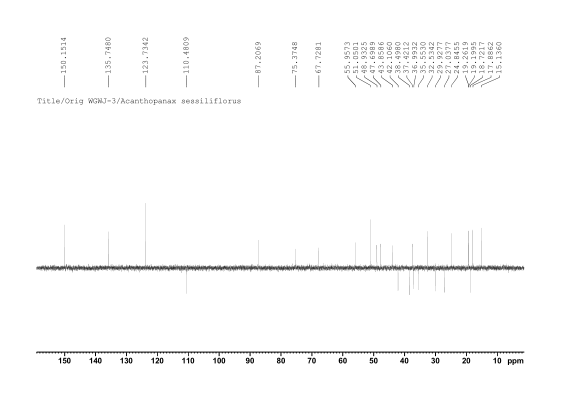


**Figure S60**. HSQC spectrum of Elesesterpene H (**8**).
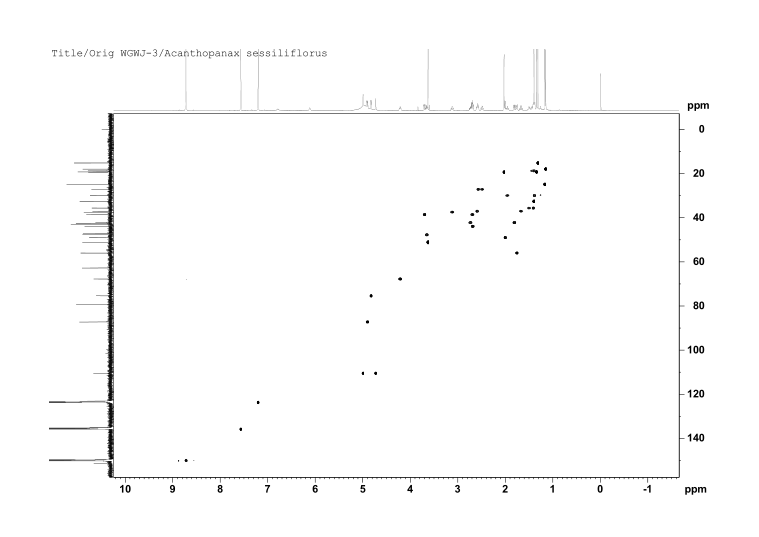


**Figure S61**. HMBC spectrum of Elesesterpene H (**8**).
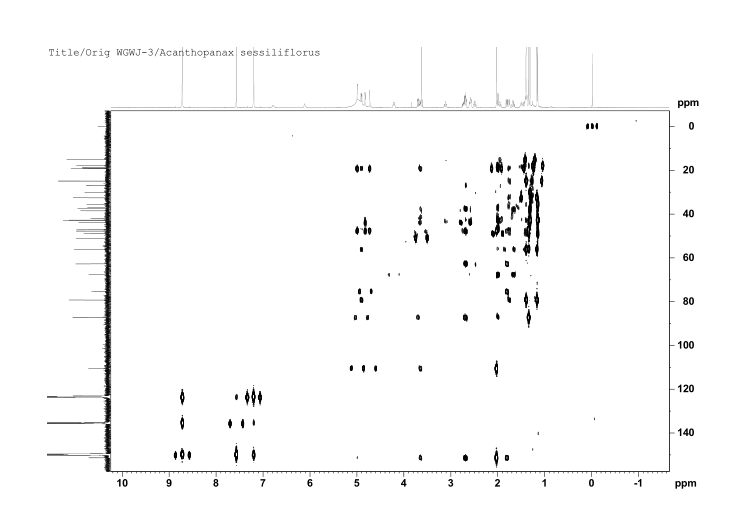


**Figure S62**. ^1^H-^1^H COSY spectrum of Elesesterpene H (**8**).
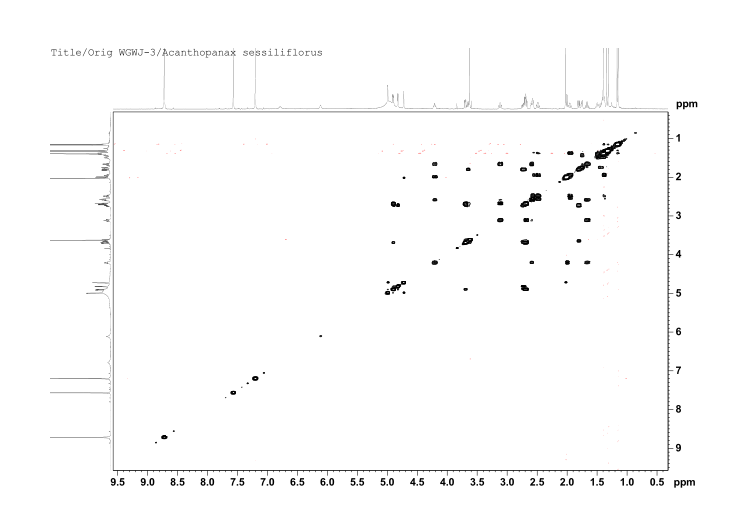


**Figure S63**. NOESY spectrum of Elesesterpene H (**8**).
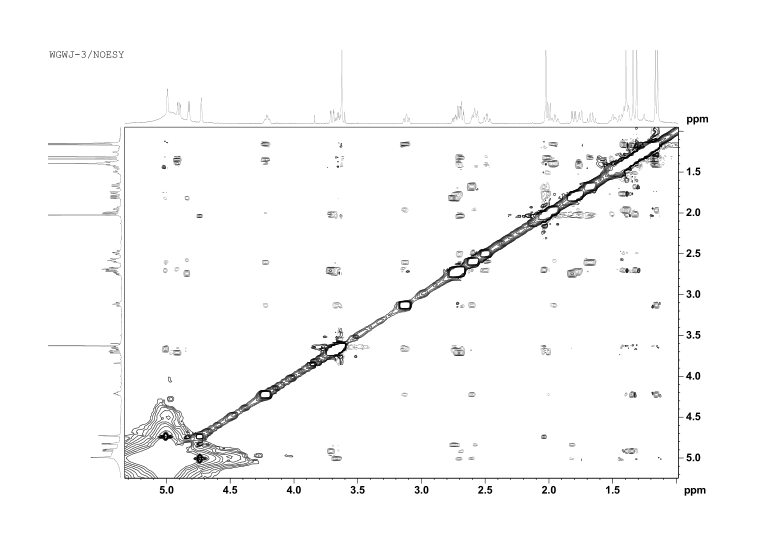


**Figure S64**. HR-ESI-MS spectrum of Elesesterpene H (**8**).

**Figure S65**. ^1^H NMR spectrum of Elesesterpene I (**9**) (600 MHz, pyridine‑*d_5_*).
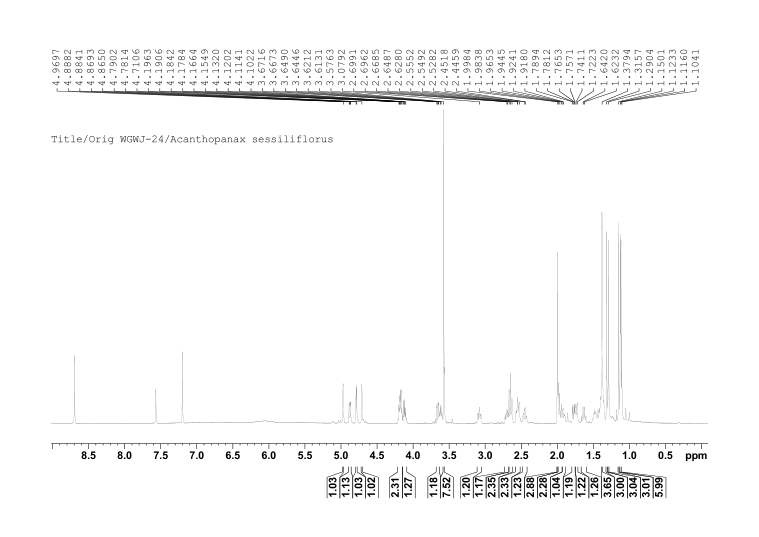


**Figure S66**. ^13^C NMR spectrum of Elesesterpene I (**9**) (150 MHz, pyridine‑*d_5_*).
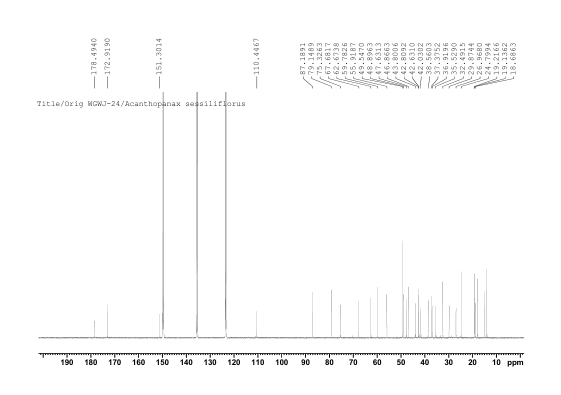


**Figure S67**. DEPT spectrum of Elesesterpene I (**9**).
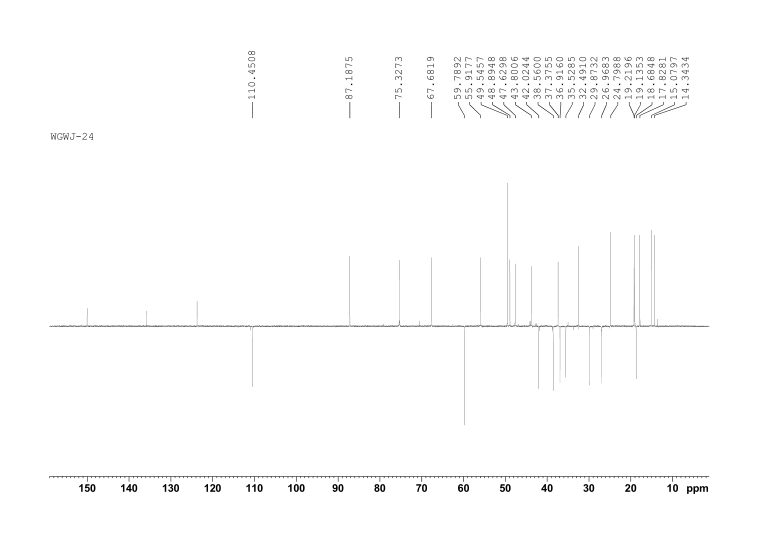


**Figure S68**. HSQC spectrum of Elesesterpene I (**9**).
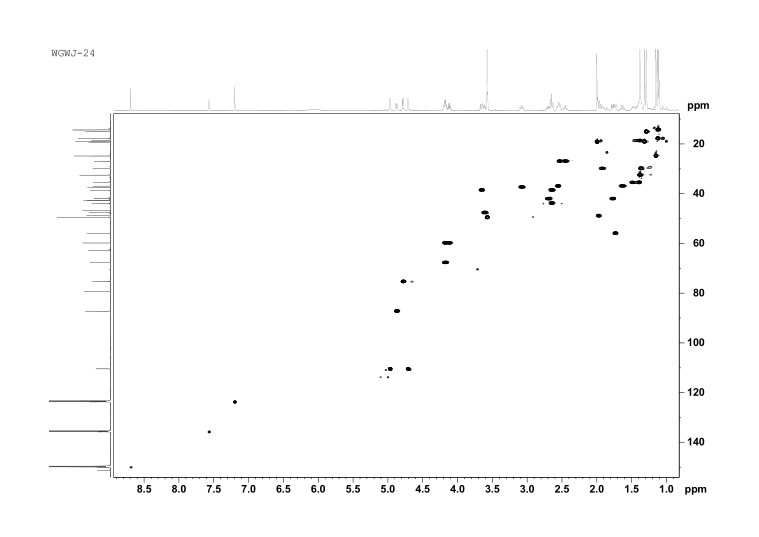


**Figure S69**. HMBC spectrum of Elesesterpene I (**9**).
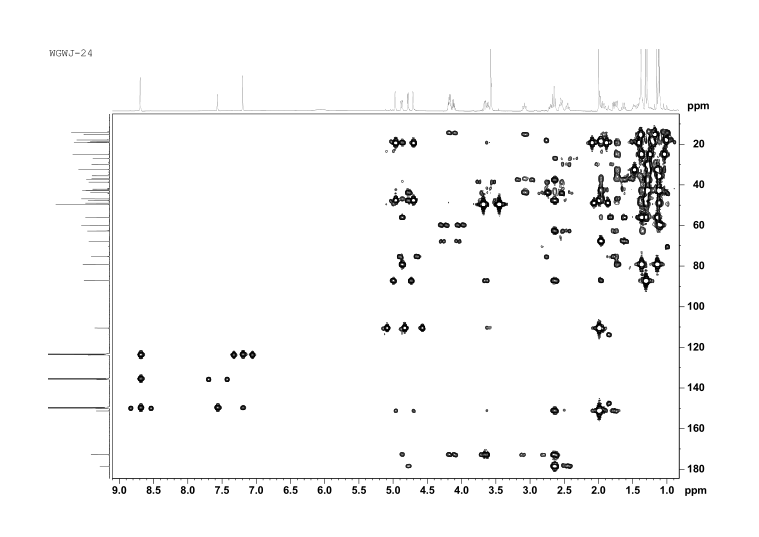


**Figure S70**. ^1^H-^1^H COSY spectrum of Elesesterpene I (**9**).
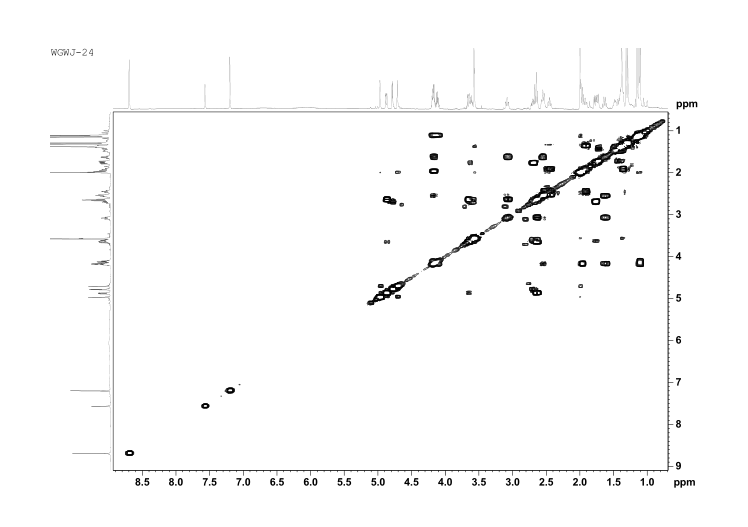


**Figure S71**. NOESY spectrum of Elesesterpene I (**9**).
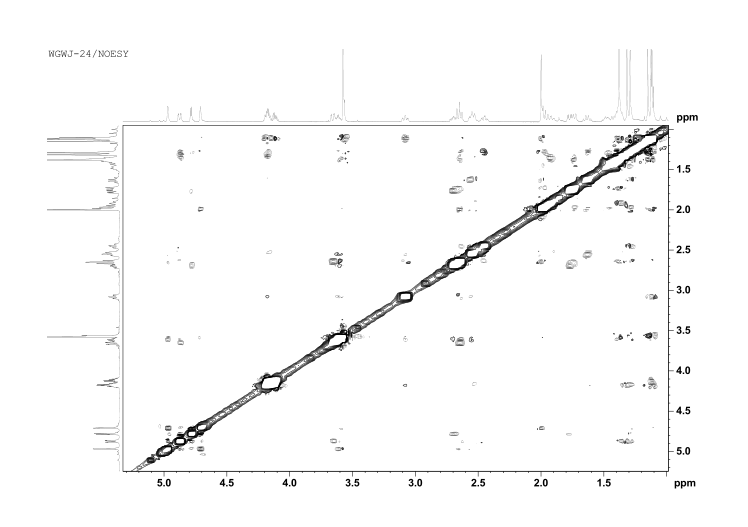


**Figure S72**. HR-ESI-MS spectrum of Elesesterpene I (**9**).

**Figure S73**. ^1^H NMR spectrum of Elesesterpene G (**10**) (600 MHz, pyridine‑*d_5_*).
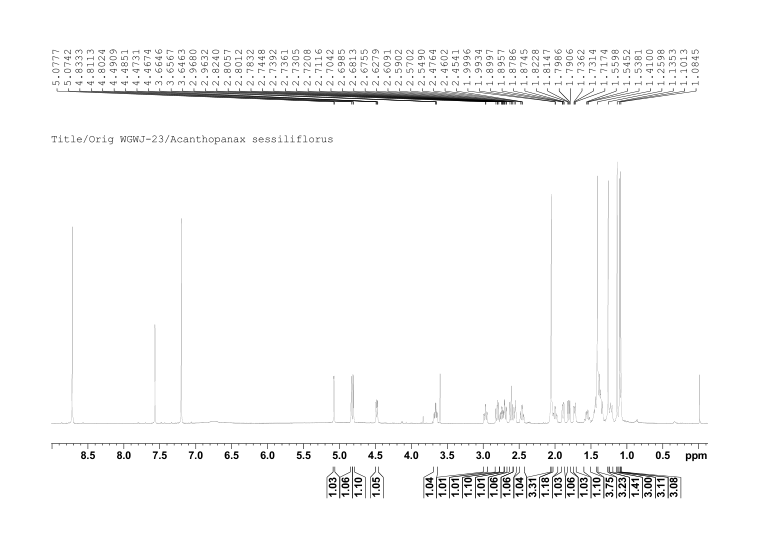


**Figure S74**. ^13^C NMR spectrum of Elesesterpene G (**10**) (150 MHz, pyridine‑*d_5_*).
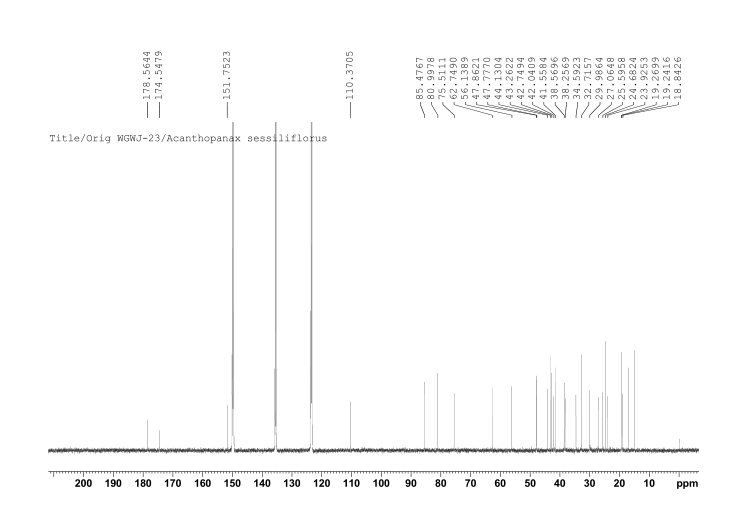


**Figure S75**. DEPT spectrum of Elesesterpene G (**10**).
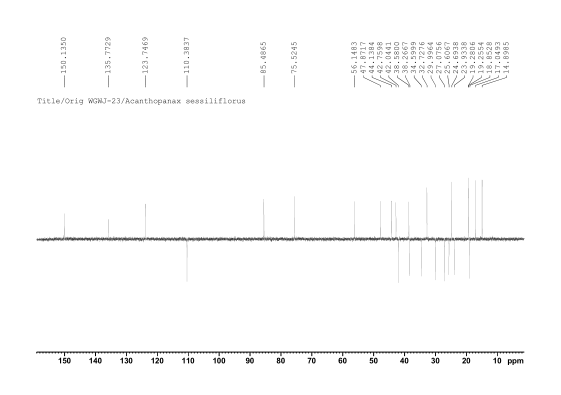


**Figure S76**. HSQC spectrum of Elesesterpene G (**10**).
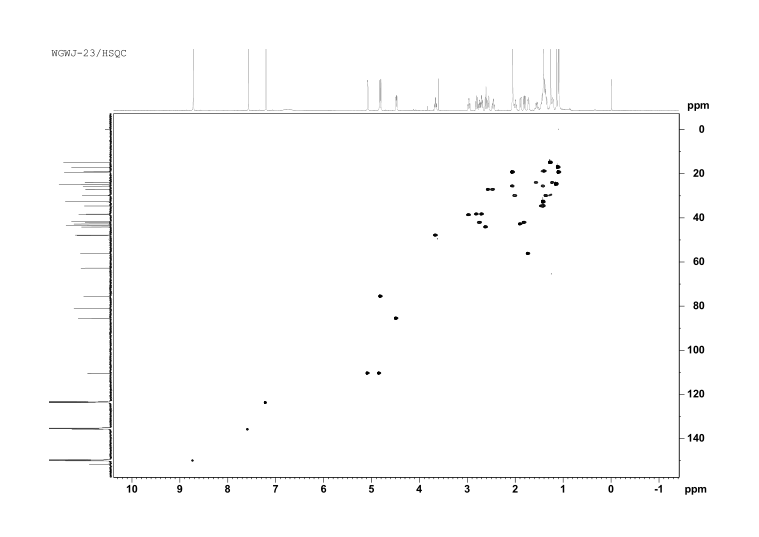


**Figure S77**. HMBC spectrum of Elesesterpene G (**10**).
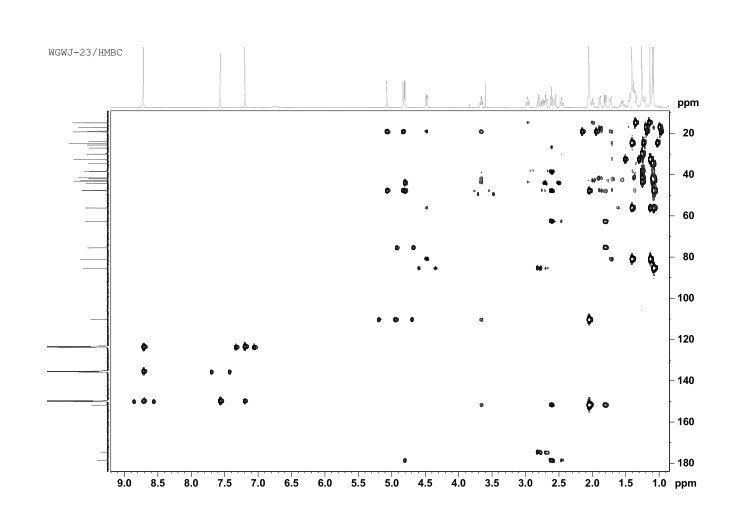


**Figure S78**. ^1^H-^1^H COSY spectrum of Elesesterpene G (**10**).
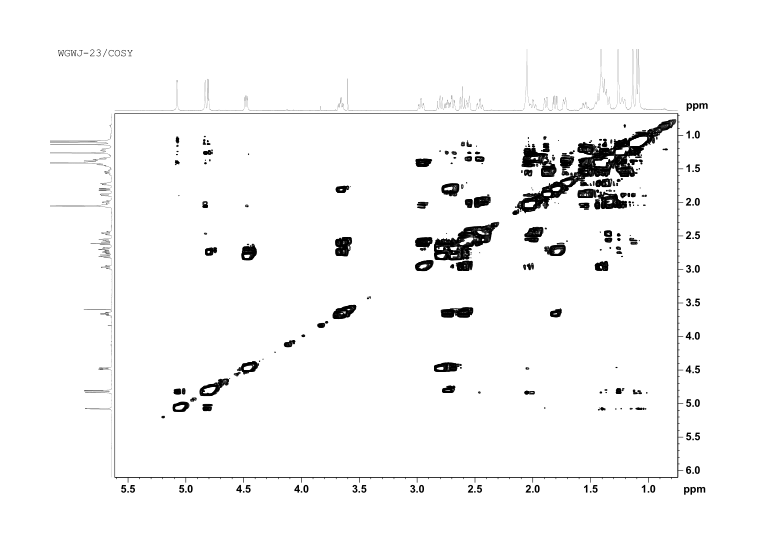


**Figure S79**. NOESY spectrum of Elesesterpene G (**10**).
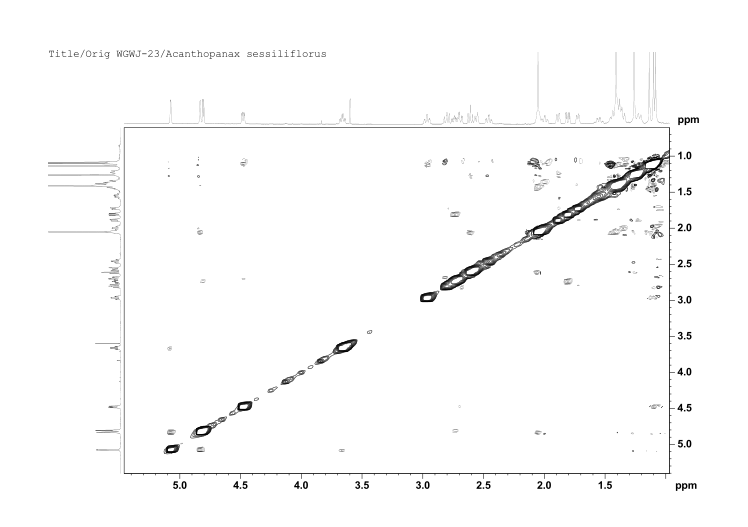


**Figure S80**. HR-ESI-MS spectrum of Elesesterpene G (**10**).

**Figure S81**. ^1^H NMR spectrum of Elesesterpene K (**11**) (600 MHz, pyridine‑*d_5_*).
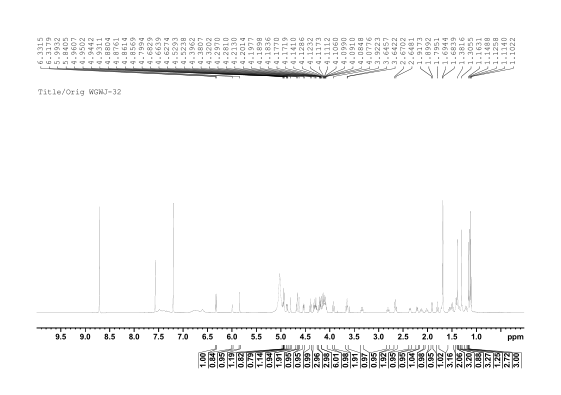


**Figure S82**. ^13^C NMR spectrum of Elesesterpene K (**11**) (150 MHz, pyridine‑*d_5_*).
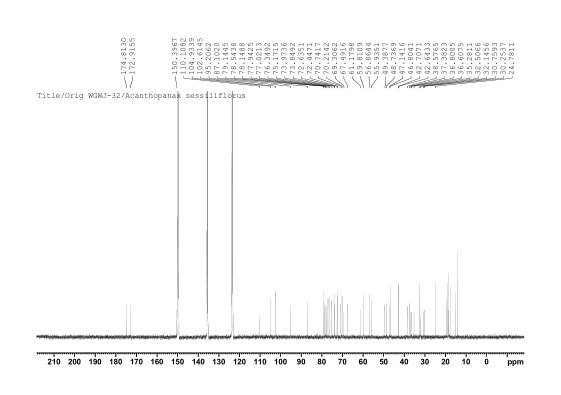


**Figure S83**. DEPT spectrum of Elesesterpene K (**11**).
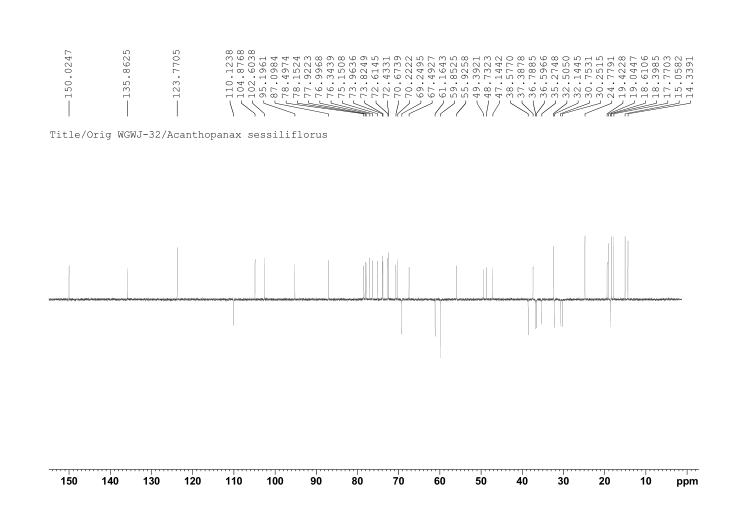


**Figure S84**. HSQC spectrum of Elesesterpene K (**11**).
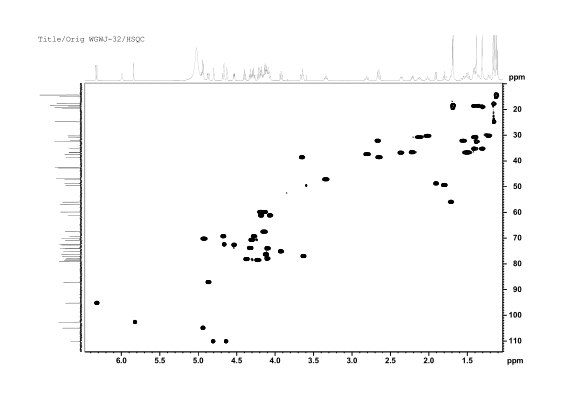


**Figure S85**. HMBC spectrum of Elesesterpene K (**11**).
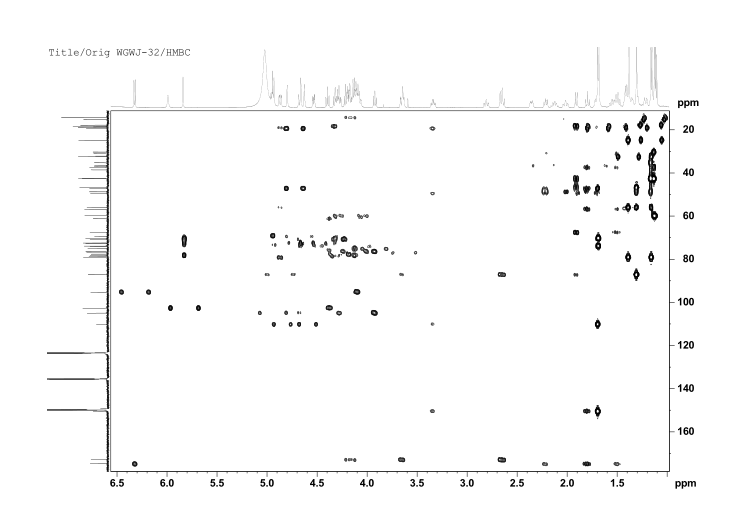


**Figure S86**. ^1^H-^1^H COSY spectrum of Elesesterpene K (**11**).
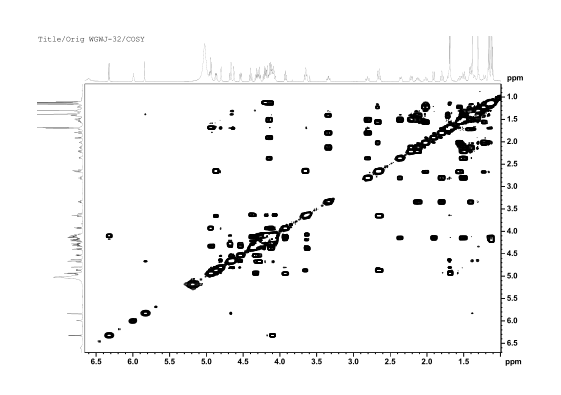


**Figure S87**. NOESY spectrum of Elesesterpene K (**11**).
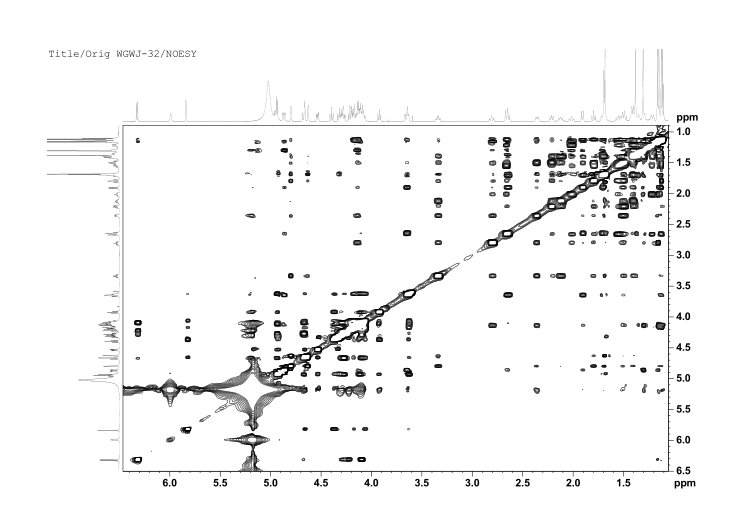


**Figure S88**. HR-ESI-MS spectrum of Elesesterpene K (**11**).

**Figure S89**. Gas chromatogram of the L-Rhamnose, D-Glucose, compounds **3**, **4**, **5**, and **11**.


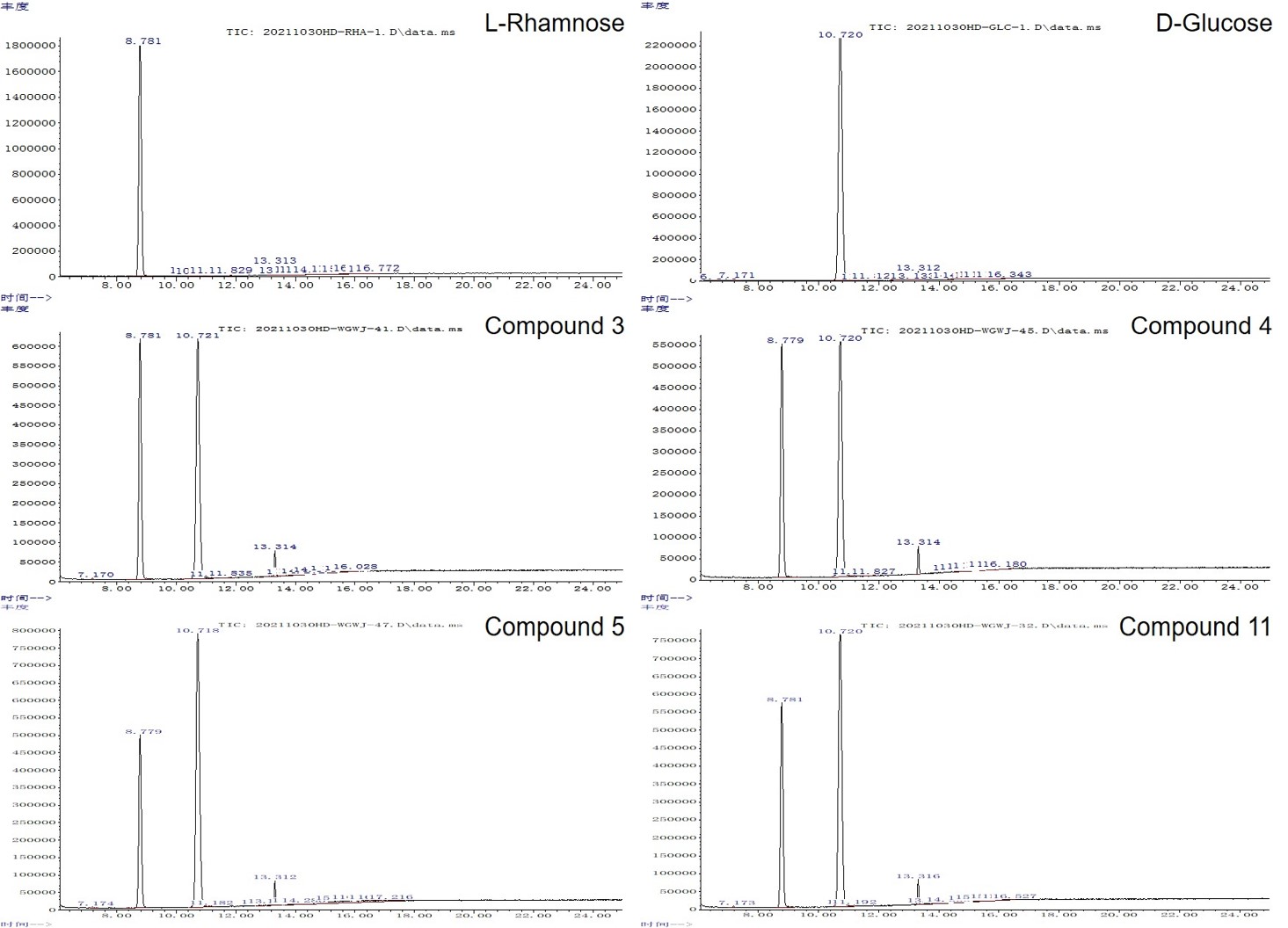

Supplement: Supplementary file 1 [file DataSheet1.docx]
